# Feedforward regulation of *Myc* coordinates lineage-specific with housekeeping gene expression during B cell progenitor cell differentiation

**DOI:** 10.1371/journal.pbio.2006506

**Published:** 2019-04-12

**Authors:** Isabel Ferreirós-Vidal, Thomas Carroll, Tianyi Zhang, Vincenzo Lagani, Ricardo N. Ramirez, Elizabeth Ing-Simmons, Alicia G. Gómez-Valadés, Lee Cooper, Ziwei Liang, Georgios Papoutsoglou, Gopuraja Dharmalingam, Ya Guo, Sonia Tarazona, Sunjay J. Fernandes, Peri Noori, Gilad Silberberg, Amanda G. Fisher, Ioannis Tsamardinos, Ali Mortazavi, Boris Lenhard, Ana Conesa, Jesper Tegner, Matthias Merkenschlager, David Gomez-Cabrero

**Affiliations:** 1 Lymphocyte Development Group, MRC London Institute of Medical Sciences, London, United Kingdom; 2 Institute of Clinical Sciences, Faculty of Medicine, Imperial College London, London, United Kingdom; 3 MRC London Institute of Medical Sciences, London, United Kingdom; 4 Gnosis Data Analysis, Heraklion, Greece; 5 Institute of Chemical Biology, Ilia State University, Tbilisi, Georgia; 6 Department of Developmental and Cell Biology and Center for Complex Biological Systems, University of California, Irvine, California, United States of America; 7 Computational Regulatory Genomics, MRC London Institute of Medical Sciences, London, United Kingdom; 8 Computer Science Department, University of Crete, Heraklion, Greece; 9 Genomics of Gene Expression Laboratory, Principe Felipe Research Center, Valencia, Spain; 10 Department of Applied Statistics, Operations Research and Quality, Universitat Politècnica de València, Valencia, Spain; 11 Unit of Computational Medicine, Department of Medicine, Solna, Center for Molecular Medicine, Karolinska Institutet, Stockholm, Sweden; 12 Sars International Centre for Marine Molecular Biology, University of Bergen, Bergen, Norway; 13 Microbiology and Cell Science Department, IFAS, University of Florida, Gainesville, Florida, United States of America; 14 Science for Life Laboratory, Solna, Sweden; 15 Biological and Environmental Sciences and Engineering Division, Computer, Electrical and Mathematical Sciences and Engineering Division, King Abdullah University of Science and Technology, Saudi Arabia; 16 Centre for Host Microbiome Interactions, Faculty of Dentistry, Oral & Craniofacial Sciences, King’s College London, London, United Kingdom; 17 Navarrabiomed, Complejo Hospitalario de Navarra (CHN), Universidad Pública de Navarra (UPNA), IdiSNA, Pamplona, Spain; The Scripps Research Institute, United States of America

## Abstract

The differentiation of self-renewing progenitor cells requires not only the regulation of lineage- and developmental stage–specific genes but also the coordinated adaptation of housekeeping functions from a metabolically active, proliferative state toward quiescence. How metabolic and cell-cycle states are coordinated with the regulation of cell type–specific genes is an important question, because dissociation between differentiation, cell cycle, and metabolic states is a hallmark of cancer. Here, we use a model system to systematically identify key transcriptional regulators of Ikaros-dependent B cell–progenitor differentiation. We find that the coordinated regulation of housekeeping functions and tissue-specific gene expression requires a feedforward circuit whereby Ikaros down-regulates the expression of *Myc*. Our findings show how coordination between differentiation and housekeeping states can be achieved by interconnected regulators. Similar principles likely coordinate differentiation and housekeeping functions during progenitor cell differentiation in other cell lineages.

## Author summary

The human body is made from billions of cells comprizing many specialized cell types. All of these cells ultimately come from a single fertilized oocyte in a process that has two key features: proliferation, which expands cell numbers, and differentiation, which diversifies cell types. Here, we have examined the transition from proliferation to differentiation using B lymphocytes as an example. We find that the transition from proliferation to differentiation involves changes in the expression of genes, which can be categorized into cell-type–specific genes and broadly expressed “housekeeping” genes. The expression of many housekeeping genes is controlled by the gene regulatory factor Myc, whereas the expression of many B lymphocyte–specific genes is controlled by the Ikaros family of gene regulatory proteins. Myc is repressed by Ikaros, which means that changes in housekeeping and tissue-specific gene expression are coordinated during the transition from proliferation to differentiation.

## Introduction

Cell proliferation, metabolic state, and differentiation are linked: proliferating progenitor cells exit the cell cycle and adjust their metabolism as they differentiate [1–3]. Mechanistically, this coordination is thought to involve mutual antagonism between cyclin-dependent kinases that promote cell-cycle entry and transcription factors that induce tissue-specific gene expression [1,2].

**Fig 1:**
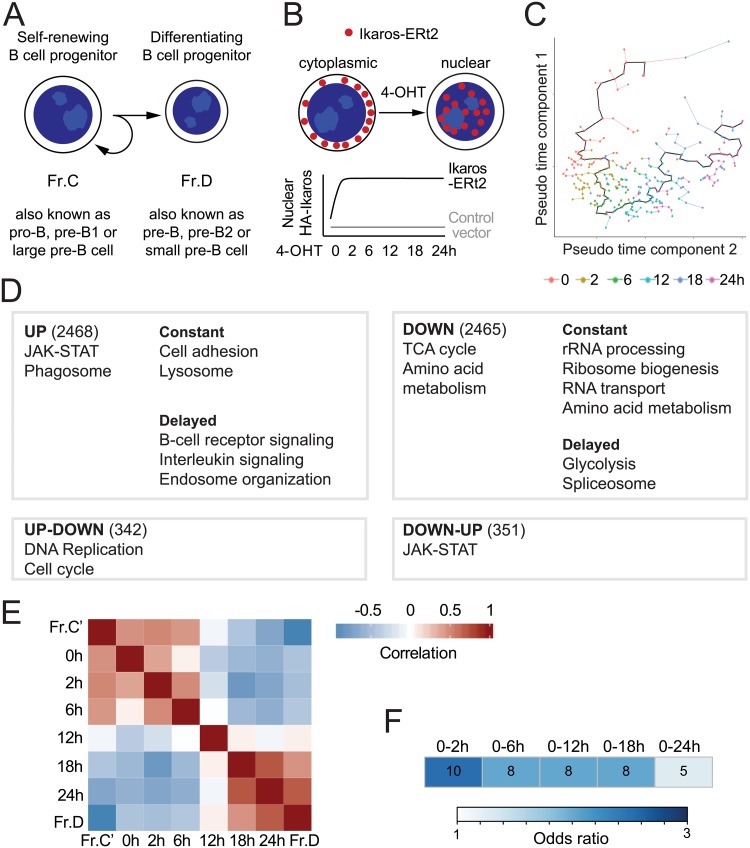
An Ikaros-driven in vitro system captures key features of in vivo B cell progenitor differentiation. (A) Schematic representation of self-renewing (left) and differentiating (right) B cell progenitors and the nomenclature used to refer to these populations in the literature. (B) Experimental design of Ikaros-induced B3 cell differentiation. RNA-seq was performed at the indicated time points. The experiment was designed so that sources of variability could be traced to library preparation and sequencing biases and corrected (see Methods). Effects of 4-OHT were monitored by addition of 4-OHT to control vector cells and found to be negligible. (C) Pseudotemporal ordering of cells as a function of their scRNA-seq transcriptomes. The pseudotime trajectory is shown in black. (D) Gene set analysis of gene expression trends in B3 differentiation. Each box contains the total number of genes and the major functional terms (*q* < 0.01) for each of 4 expression trends (up-regulated, down-regulated, first up- then down-regulated, and first down- then up-regulated). Up- and down-regulated trends are further separated into 2 subtrends, constant and delayed. (E) Comparison of the transcriptomes of the B3 in vitro differentiation time course with in vivo B cell progenitor differentiation stages. The 0 to 6 h time points resemble Fr.C, whereas 18 and 24 h are similar to Fr.D, and the 12 h time point represents a tipping point. The source of the numerical data underlying this figure is listed in S1 Data. (F) Comparison of genes differentially expressed in IKZF1 mutated human B-ALL and the B3 differentiation. Numbers denote common genes that are differentially expressed in *IKZF1-*mutated versus *IKZF1* wild-type human B-ALL and the top 200 genes regulated at the indicated times after Ikaros induction in B3 cells. The color scale indicates the odds ratio for the enrichment of Ikaros-regulated gene during B3 cell differentiation among differentially expressed genes in human B-ALL with and without *IKZF1* mutations. B-ALL, B cell progenitor acute lymphoblastic leukemia; ERt2; Fr.C, proliferating B cell progenitor; Fr.D, differentiating B cell progenitor; HA, haemagglutinin; JAK-STAT, Janus kinase/signal transducers and activators of transcription; RNA-seq, RNA sequencing; scRNA-seq, single cell RNA sequencing; TCA, tricarboxylic acid cycle; 4-OHT, 4-hydroxy-tamoxifen.

A detailed inventory of differentiation stages is available for mammalian haematopoiesis. In B cell differentiation, discrete stages are defined by CD markers [4], gene expression profiles [5] (www.immgen.org), transcription factor binding [6–8], and cell-cycle states [9–11]. A critical step is the transition of proliferating B cell progenitors towards cell-cycle arrest and differentiation. We refer to proliferating B cell progenitors as Fr.C following Hardy’s nomenclature [4]; this stage is also known as the pro-B, pre-B1, or large pre–B cell stage (Fig 1A). We refer to quiescent, differentiating B cell progenitors as Fr.D following Hardy’s nomenclature [4]; this stage is also known as the pre-B, pre-B2, or small pre–B cell stage (Fig 1A). Transcriptional regulators of the Ikaros family of zinc finger proteins are up-regulated at this transition [12] and are required for B cell progenitor differentiation in vivo [13–15]. *IKZF1*, the gene encoding Ikaros, is recurrently mutated in human B cell progenitor acute lymphoblastic leukemias (B-ALLs) with translocations between the *IGH* locus and the *ABL1* proto-oncogene (BCR-ABL1) [16,17].

Here, we employ an inducible system in a B cell progenitor line that models the transition from B cell progenitor proliferation toward cell-cycle arrest and differentiation upon the regulated delivery of the transcription factor Ikaros from the cytoplasm into the nucleus [7,18]. The availability of this in vitro model, combined with access to primary B cell progenitors for validation experiments, makes B cell progenitor differentiation an attractive system to study progenitor differentiation and the mechanisms that link differentiation with changes in cell cycle and metabolism. To understand the regulatory control of B cell progenitor differentiation, we developed an algorithm that examines the temporal correlation between the expression of transcription factors and their target genes over the course of progenitor differentiation and scores the relative contribution of different transcription factors This approach, which is transferable to other cell-state transitions, highlighted that the Ikaros targets Foxo1 and Myc as high-scoring transcription factors. Perturbation experiments showed that the transcriptional repression of *Myc* was critical for the coordinated regulation of lineage-specific, cell-cycle, and metabolic genes. Ikaros-mediated repression of *Myc* connects B cell differentiation to cell-cycle exit and metabolic adaptation. Similar principles may coordinate differentiation and housekeeping functions during progenitor differentiation in other cell lineages.

## Results

### Temporal dissection of gene expression changes during B cell progenitor differentiation

We controlled the dosage of nuclear Ikaros-ERt2 with temporal precision by the addition of 4-hydroxy-tamoxifen (4-OHT) in the pre–B cell line B3 [7,18]. This model is designed to approximate the B cell linker (BLNK)-dependent increase of *Ikzf3* expression in primary B cell progenitors in response to pre-B cell receptor signaling [12], and the nuclear translocation of Ikaros recapitulates the majority of gene expression changes that distinguishes proliferating (Fr.C) from differentiating (Fr.D) B cell progenitors in vivo [7,18]. We performed time-resolved RNA sequencing (RNA-seq) of 4-OHT-treated B3 cells expressing Ikaros-ERt2 or control vector (Fig 1B) at 6 time points after 4-OHT. We combined pairwise comparison between time points by limma [19] with time-course analysis by maSigPro [20] to identify 5,865 differentially expressed genes (S1 Table). Pseudotemporal ordering [21] of single cell RNA-seq (scRNA-seq) data showed an unbranched path over the experimental differentiation time course (Fig 1C).

### Dynamics and validation of the experimental system

Gaussian mixture modeling for model-based clustering [22] resolved up- and down-regulated genes, subtrends of immediate and delayed regulation, and 2 nonmonotonic up-down–and down-up–regulated groups (Fig 1D). Functional characterization of these trends by gene set analysis showed that up-regulated genes were enriched in Janus kinase/signal transducers and activators of transcription (JAK-STAT) signaling, cell adhesion, and B cell receptor and interleukin signalling, while metabolic functions, RNA metabolism, and ribosome biogenesis were enriched among down-regulated genes. This analysis indicates a clear distinction between induced (signaling and cell–cell communication) and repressed (mainly metabolism-related) processes during B cell progenitor differentiation (Fig 1D).

To ask how well this experimental system models B cell progenitor differentiation in vivo, we compared dynamic gene expression at each time point with static, developmental stage-specific gene expression by primary B cell progenitors [5]. Up to 6 h after 4-OHT-induced nuclear translocation of Ikaros, gene expression correlated positively with the less mature Fr.C and negatively with the more mature Fr.D states in vivo (Fig 1E). The 12 h time point marked a tipping point at which the positive correlation with Fr.C and the negative correlation with Fr.D was lost (Fig 1E). After that, gene expression correlated positively with the more mature Fr.D and negatively with the less mature Fr.C (Fig 1E). Therefore, gene expression in B3 cells showed a transition from an Fr.C-like state to an Fr.D-like state within a 24-h time frame. The experimental system allowed the capture not only of the start and end points but also the dynamics that occurred between them.

To examine the ability of this model system to pinpoint Ikaros target genes relevant to human disease, we assembled gene expression profiles of 1,404 B-ALL samples with and without *IKZF1* mutations (S1 Fig and associated text). Genes that were differentially expressed between Fr.C and Fr.D were preferentially deregulated in *IKZF1*-mutated B-ALL [23,24], including current and potential therapeutic targets [25–27] and prognostically relevant gene signatures in B-ALL [17,28] (S1 Fig). There was significant overlap between differential gene expression in *IKZF1-*mutated B-ALL and early gene expression changes in B3 cells at 0 to 2 h after Ikaros induction (Fig 1F, odds ratio = 2.53, adjusted [adj.] *P* = 0.02 for the 200 top differentially expressed genes). Because early gene expression changes are likely direct, this finding indicates that our in vitro system identifies gene expression changes in *IKZF1*-mutated B-ALL that may result directly from the loss of Ikaros function.

### The progression from proliferating to resting stages of pre–B cell differentiation is marked by “reverse” metabolic reprogramming

**Fig 2:**
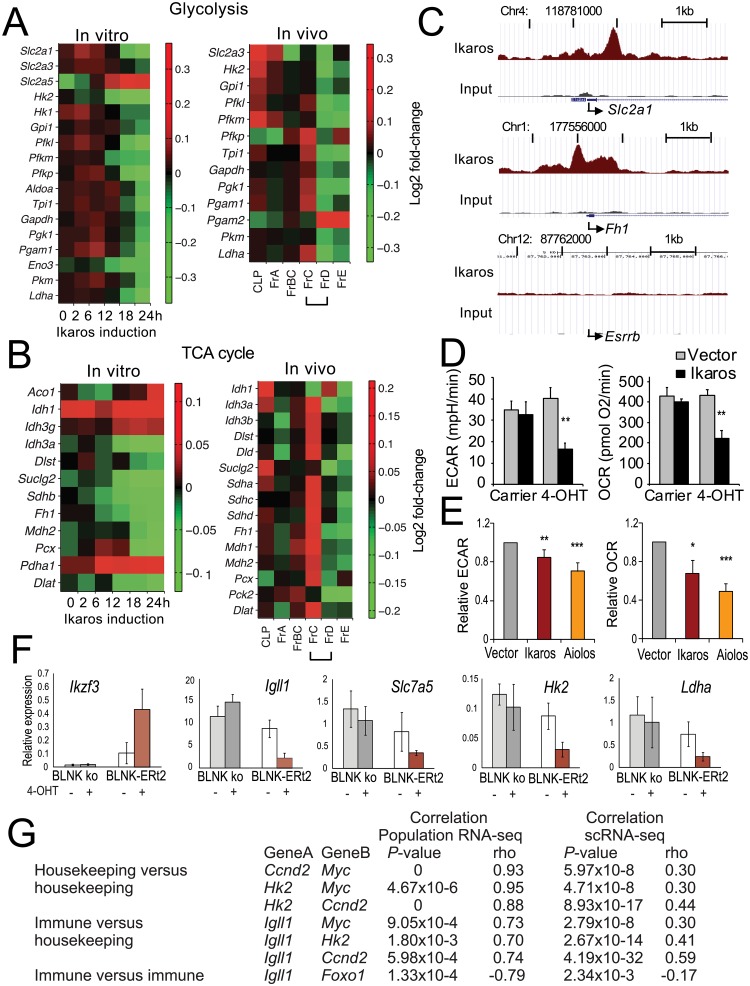
Progression from proliferating to resting stages of pre–B cell differentiation is marked by metabolic reprogramming. (A) Heat map of glycolysis gene expression in B3 cells (left) and at consecutive stages of B cell progenitor differentiation in vivo (right). The transition from Fr.C to Fr.D is marked by a bracket. The source of the numerical data underlying this figure is listed in S1 Data. (B) Heat map of TCA cycle gene expression in B3 cells (left) and at subsequent stages of B cell progenitor differentiation in vivo (right). The source of the numerical data underlying this figure is listed in S1 Data. (C) Ikaros ChIP-seq shows that Ikaros targets metabolic genes. The embryonic stem cell gene *Esrrb* is a negative control. Other Ikaros-bound core metabolic genes are *Dlat*, *Hk2*, *Idh3g*, *Slc2a3*, *Dlst*, *Sdhb*, *Pgam1*, *Mdh2*, *Pfkl*, *Dld*, *Pfkp*, *Sdha*, *Pcx*, *Idh1*, *Fh1*, *Aco1*, *Slc2a1*, *Slc2a5*, *Aldoa*, *Pdha1*, *Pkm*, *Pfkm*, *Gpi1*, *Pck2*, *Eno3*, *Suclg*, and *Pgk1*. (D) Ikaros reduces ECAR, a measure of glycolysis (left) and the OCR (right) in B3 cells after 24 h (mean ± SEM of 3 biological replicates, **P < 0.005, two-*tailed t* test). The numerical data underlying this figure are included in S1 Data. (E) Ikaros and the Ikaros family member Aiolos reduce ECAR and OCR in primary B cell progenitors (mean ± SEM of 6 to 7 biological replicates, *P < 0.05, **P < 0.005, ***P < 0.0001, one-tailed *t* test). The numerical data underlying this figure are included in S1 Data. (F) Impact of BLNK-mediated induction of *Ikzf3* on the expression of metabolic genes. The numerical data underlying this figure are included in S1 Data. (G) The regulation of immune and housekeeping genes is coordinated at the level of cell populations and in single cells. BLNK, B cell linker; ChIP-seq, chromatin immunoprecipitation sequencing; ECAR, extracellular acidification; Fr.C, proliferating B progenitor; Fr.D, differentiating B cell progenitor; OCR, oxygen consumption rate; TCA cycle, tricarboxylic acid cycle.

To follow up on the down-regulation of genes related to metabolism (Fig 1D), we compared the expression of glycolysis and tricarboxylic acid (TCA) cycle genes during B3 cell differentiation in vitro (Fig 2A and 2B, left) and B cell progenitor differentiation in vivo (Fig 2A and 2B, right). Key glycolysis and TCA cycle genes were down-regulated in vitro and in vivo (Fig 2A and 2B; the transition from Fr.C to Fr.D is indicated with a bracket). Analysis of Ikaros chromatin immunoprecipitation sequencing (ChIP-seq) data [7] showed that numerous core metabolic genes were directly bound by Ikaros, as illustrated by the glucose transporter *Slc2a1* and the TCA cycle gene *Fh1* (Fig 2C; see figure legend for a list of Ikaros-bound core metabolic genes).

In extracellular flux assays, nuclear translocation of Ikaros triggered a pronounced reduction (55%–65%) in extracellular acidification (ECAR) as a measure of lactate production (Fig 2D, left). The oxygen consumption rate (OCR) was reduced by 45%–50% (Fig 2D, right). This was accompanied by reduced mechanistic target of rapamycin complex 1 (mTORC1) activity, as read out by phosphorylation of S6 ribosomal protein (S3A Fig) and the transcriptional up-regulation of autophagy genes (S3B Fig).

To validate these findings, we transduced primary Fr.C-like B cell progenitors with Ikaros or *Ikzf3* (Aiolos) and determined the resulting changes in ECAR and the OCR. Ikaros and Aiolos reduced the ECAR and the OCR of primary B cell progenitors (Fig 2E).

In the experiments described above, changes in metabolic gene expression and metabolic activity were induced by the expression of *Ikzf1* or *Ikzf3*. During B cell progenitor differentiation, *Ikzf3* expression is initiated by signaling through the pre-B cell receptor via a pathway that requires BLNK [12]. To determine whether pre-B cell receptor signaling is linked to metabolic regulation, we used an experimental system in which BLNK activity can be inducibly restored in BLNK-deficient B cells by means of a 4-OHT-inducible BLNK-ERt2 fusion protein [29]. As expected, restoration of BLNK activity induced *Ikzf3* and repressed *Igll1*, a known target of *Ikzf1* and *Ikzf3* during the the Fr.C to Fr.D transition [12,18] (Fig 2F, top). Restoration of BLNK activity led to the repression of the metabolic genes *Slc7a5*, *Hk2*, and *Ldha* (Fig 2F, bottom), indicating that pre-B cell receptor signaling controls the expression of metabolic genes in B cell progenitors.

These data demonstrate altered metabolic gene expression, reduced glycolytic flux, and a drop in oxygen consumption at the transition of B cell progenitors from proliferation to quiescence in vitro and in vivo. This indicates “reverse” metabolic reprogramming towards a less glycolytic state [30,31]. The expression of B cell genes such as *Igll1* and *Foxo1* was correlated with the expression of housekeeping genes, such as *Myc*, the metabolic gene *Hk2*, and the cell-cycle gene *Ccnd2*, not only at the level of cell populations but also in individual cells (Fig 2G). We conclude that the regulation of B cell–specific genes is coordinated with the regulation of housekeeping pathways during B cell progenitor differentiation.

### Systematic identification of key transcription factors in B cell progenitor differentiation

**Fig 3:**
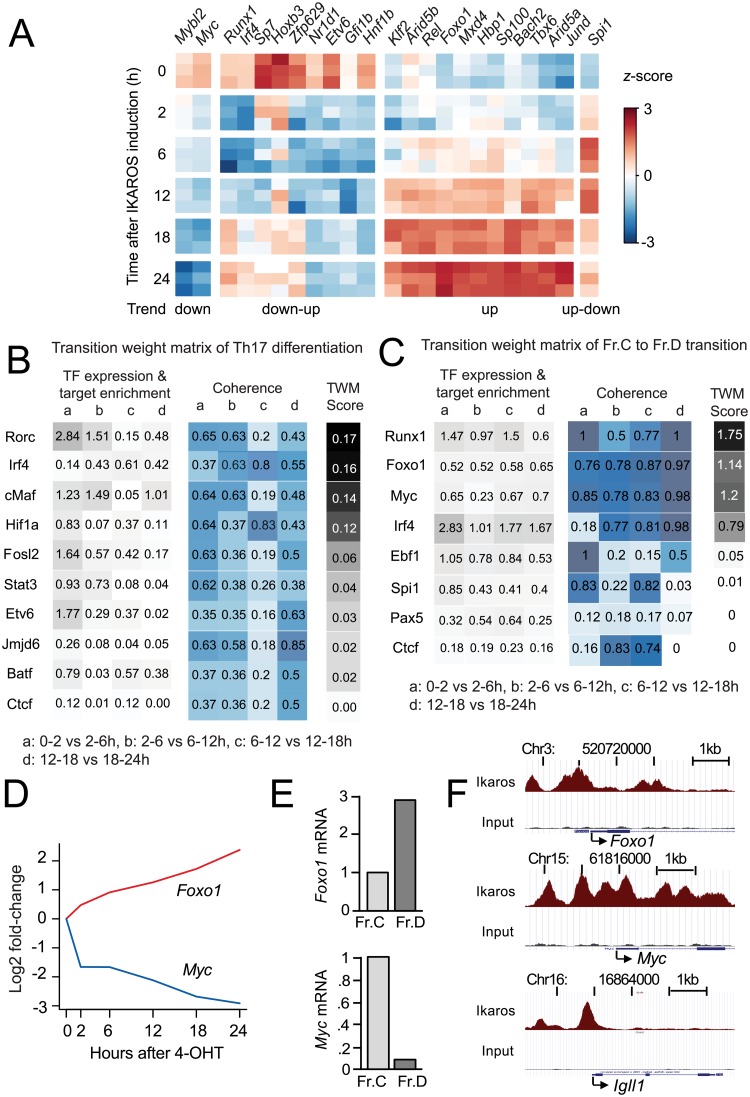
Systematic identification of candidate regulators of B cell progenitor differentiation. (A) Heat map of 23 candidate transcriptional regulators of B cell progenitor differentiation identified by 3 RNA-seq replicates at the indicated time after Ikaros induction in B3 cells; expression trends are indicated. The source of the numerical data underlying this figure is listed in S1 Data. (B) TWM analysis of Th17 differentiation. See text and S3 Fig for details and “Methods” for a comprehensive mathematical description. (C) TWM analysis of the Fr.C to Fr.D transition based on target genes defined by ChIP-seq identified RUNX1, FOXO1, and Myc as high-scoring regulators. (D) Progressive up-regulation of *Foxo1* mRNA and down-regulation of *Myc* mRNA with time after Ikaros induction. Shown is the mean of 3 independent RNA-seq replicates. (E) Up-regulation of *Foxo1* mRNA during in vivo B cell progenitor differentiation ([5]; www.immgen.org). Down-regulation of *Myc* mRNA during in vivo B cell progenitor differentiation ([5]; www.immgen.org). (F) Ikaros ChIP-seq [7] shows Ikaros binding to the promoter regions of *Foxo1* and *Myc*. The established Ikaros target gene *Igll1* is shown for reference. ChIP-seq, chromatin immunoprecipitation sequencing; FOXO1, Forkhead Box O1; Fr.C, proliferating B cell progenitor; Fr.D, differentiating B cell progenitor; Myc, MYC proto-oncogene; RNA-seq, RNA sequencing; RUNX1, RUNT-related transcription factor; Th17, T helper 17; TWM, transition weight matrix.

B cell progenitor differentiation is marked by the differential expression of numerous transcription factors. To define the key transcription factors and the regulatory pathways involved in this process, we first identified all transcription factor genes that showed a significant and robust (log_2_ fold change > 1.5) change in expression between consecutive time points. We considered transcription factors that were differentially expressed (adj. *P* < 0.01) between Fr.C and Fr.D in vivo ([5]; www.immgen.org). We included transcription factors that showed up-down–or down-up–regulation over the in vitro time course, based on the consideration that genes with nonmonotonic expression may be important for B cell progenitor differentiation even if they were not differentially expressed between the start and the end of the transition. This resulted in a total of 23 candidate transcriptional regulators (Fig 3A, Table 1). To evaluate the potential importance (or “weight”) of transcriptional regulators during cell-state transitions, we developed an approach that numerically integrates the differential expression over time of transcription factors and their target genes, which we refer to as transition weight matrix (TWM). For each transcription factor, we identified potential target genes based on transcription factor ChIP-seq peaks in gene promoters. We then determined the enrichment of transcription factor binding over differentially expressed genes and multiplied this enrichment with the log_2_ fold change in transcription factor mRNA expression for each time interval. The resulting values were summed over the time series to yield a score for transcription factor expression and target enrichment over time. We next examined to what extent the expression of each transcription factor correlated with mRNA levels of its target genes in a consistent fashion over time, which we term “coherence.” Coherence between the expression of each transcription factor and its target genes was determined for each time interval and summed over the time series to yield a score for coherence. Finally, the score for transcription factor expression and target enrichment over time was multiplied with the score for coherence to yield a TWM score for each transcription factor. Details of this approach as well as a comprehensive mathematical description are provided in S3 Fig and in the “Methods” section.

**Table 1:** Differentially expressed transcription factors during B cell progenitor differentiation.

Factor	ChIP-seq target no.	DNase-seq target no.	Inclusion criteria	Direction	In vivo log_2_ FC	In vivo adj. *P*	Ikaros target*	DNase-seq TWM score
Arid5a	N/A	1516	In vivo	UP	1.043	0.006	1	1.808
Foxo1	2186	1154	In vivo	UP	1.533	0.005	1	1.668
Mxd4	N/A	3192	In vivo	UP	1.618	0.005	1	1.578
Hbp1	N/A	2312	In vivo	UP	1.426	0.003	1	1.530
Sp100	N/A	7065	In vivo	UP	1.268	0.004	0	1.414
Mybl2	N/A	2178	In vivo	DOWN	−1.025	0.005	1	0.902
Bach2	N/A	3338	In vivo	UP	0.945	0.003	1	0.794
Jund	N/A	2271	In vivo	UP	2.177	0.003	1	0.426
Spi1	8572	4865	In vitro	UP-DOWN	0.656	0.184	1	0.282
Myc	9269	3594	In vivo	DOWN	−3.426	0.001	1	0.240
Irf4	6237	2705	In vitro	DOWN-UP	0.631	0.108	1	0.135
Nr1d1	N/A	3329	In vitro	DOWN-UP	−0.108	0.743	1	0.112
Runx1	215	4686	In vitro	DOWN-UP	0.041	0.882	1	0.050
Rel	N/A	1327	In vivo	UP	0.868	0.003	1	0.026
Sp7	N/A	4128	In vitro	DOWN-UP	0.130	0.533	0	0.016
Etv6	N/A	4253	In vitro	DOWN-UP	−0.038	0.887	1	0.009
Gfi1b	N/A	1284	In vitro	DOWN-UP	−0.561	0.064	1	0.008
Klf2	N/A	5218	In vivo	UP	1.825	0.009	1	0.007
Arid5b	N/A	1126	In vivo	UP	0.771	0.009	1	0.004
Hnf1b	N/A	461	In vitro	DOWN-UP	0.022	0.939	1	0.000
Tbx6	N/A	8	In vivo	UP	1.279	0.003	1	0.000
Zfp629	N/A	264	In vitro	DOWN-UP	0.350	0.157	1	0.000
Hoxb3	N/A	10	In vitro	DOWN-UP	0.126	0.695	0	0.000

“In vivo” denotes differentially expressed between Fr.C and Fr.D B cell progenitors in vivo [5]. “In vitro” denotes differentially expressed during the time course of B3 cell differentiation. “Direction” denotes up- or down-regulation of the transcription factor.

*With the exception of Hoxb3, Sp7, and Sp100, the promoters of all 23 transcription factors are directly bound by Ikaros ChIP-seq peaks [7].

**Abbreviations**: adj., adjusted; ChIP-seq, chromatin immunoprecipitation sequencing; DNase-seq, DNase I hypersensitive sites sequencing; FC, fold change; Fr.C, proliferating B cell progenitor; Fr.D, differentiating B cell progenitor; N/A, not applicable; TWM, transition weight matrix.

We validated the TWM approach using the paradigm of T helper 17 (Th17) T-cell differentiation, in which the key transcriptional drivers are known [32,33] and high-quality transcription factor ChIP-seq data are available (S2 Table). TWM correctly identified RORC (TWM score = 0.17), the signature transcription factor of Th17 cells, and IRF4 (TWM score = 0.14), which is required for *Rorc* expression, as transcription factors with the highest TWM score (Fig 3B). c-Maf (*Maf*; TWM score = 0.14), a transcription factor with an established role in *Rorc* induction and Th17 differentiation [34], and Hif1-alpha (*Hif1a*; TWM score = 0.12), which controls the balance between Th17 and regulatory T (Treg) cell differentiation [35,36], also scored highly. Therefore, TWM successfully identified key regulators of Th17 cell differentiation.

To apply TWM to B cell progenitor differentiation, we retrieved published ChIP-seq data sets for transcription factors in B cell progenitors. ChIP-seq data for the transcription factors Runx1, Foxo1, Myc, Irf4, Spi1, and Jund, but ChIP-seq data for Jund did not contain statistically significant peaks by CLCbio Peak Finder [37]. This left Runx1, Foxo1, Myc, Irf4, and Spi1 as potential regulators for which potential target genes could be identified based on promoter-proximal ChIPseq peaks (Table 1; proximal genes were identified by RGMatch [38]). We included EBF1 and Pax5 as key factors of known importance for B cell differentiation and CTCF as a negative control. Runx1 (TWM score = 1.75), Myc (TWM score = 1.20), and Foxo1 (TWM score = 1.14) were identified as highly ranked transcription factors (Fig 3C).

*Runx1* showed transient down-regulation followed by up-regulation. RUNX1 ChIP-seq target genes showed enrichment for differential expression (*P* < 0.0005) and good coherence (Fig 3C). *Foxo1* was progressively up-regulated and showed good enrichment for differential expression of its ChIP-seq target genes (*P* < 0.001). Promoters bound by FOXO1 were mainly up-regulated (*P* < 10 × 10^−36^). *Myc* was progressively down-regulated and also showed strong enrichment for differential expression of its ChIP-seq target genes (*P* < 0.001). Promoters bound by Myc were mainly down-regulated (*P* < 10 × 10^−9^). The relevance of FOXO1 and Myc for primary B cell progenitor differentiation is supported by the up-regulation of *Foxo1* mRNA and the down-regulation of *Myc* mRNA during in vivo B cell progenitor differentiation (Fig 3E) and direct binding of Ikaros to the *Foxo1* and *Myc* promoters in B cell progenitors (Fig 3F).

For cell-state transitions for which sufficient ChIP-seq data are not available, TWM can be implemented based on promoter accessibility and the presence of transcription factor motifs in target gene promoters. We used DNase I hypersensitive sites sequencing (DNase-seq) to assess promoter accessibility at each time interval and the presence of transcription factor motifs for all 23 differentially expressed transcription factors (Table 1, Fig 3A). TWM identified Spib (TWM score = 2.62), Arid5a (TWM score = 1.81), Foxo1 (TWM score = 1.67), and the Myc antagonist Mxd4 (TWM score = 1.58) as top-scoring transcription factors (Table 1).

For validation of the TWM results, we focused on Foxo1 and Myc for two reasons. First, Foxo1 and the Myc pathway scored highly in both ChIP-seq and DNase-seq–based TWM approaches. Second, *Foxo1* and *Myc* show monotonic changes in expression (up- and down-regulation, respectively) during the B3 cell differentiation time course, which facilitates the analysis of their impact on B cell progenitor differentiation.

### Transcriptional up-regulation of *Foxo1* is linked to FOXO1 target gene expression during B cell progenitor differentiation

**Fig 4:**
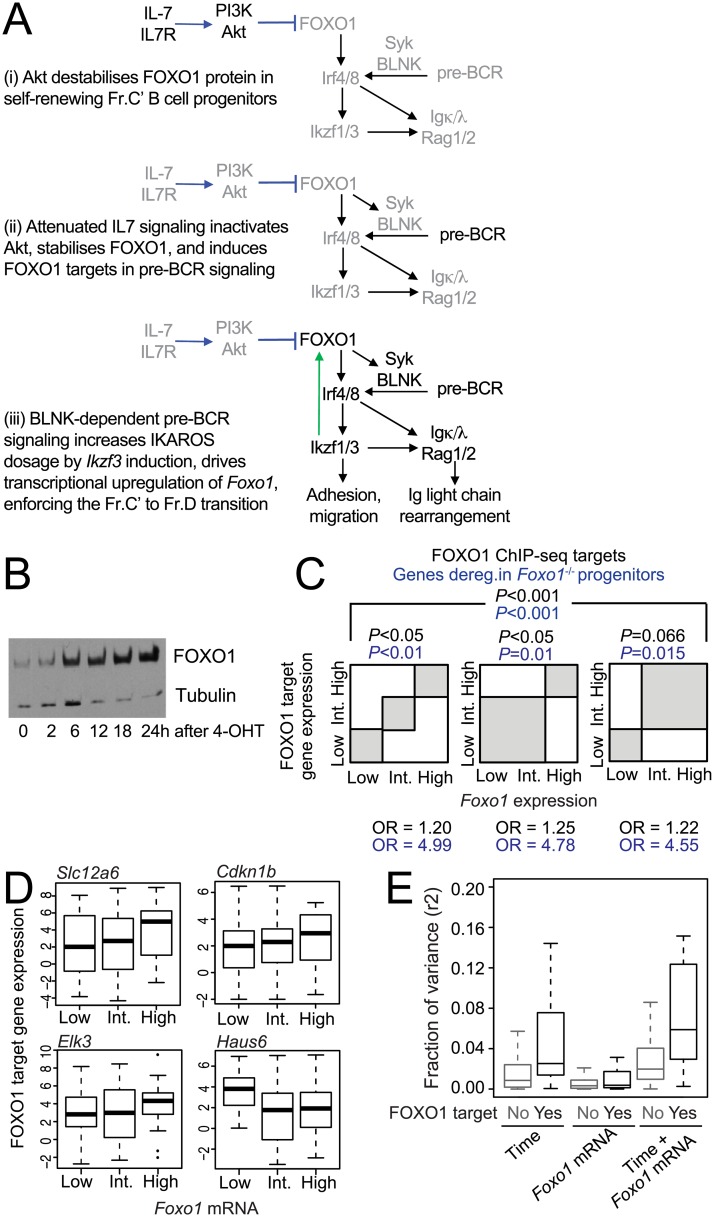
Transcriptional regulation of FOXO1 target genes. (A) Scheme of post-translational (i and ii) and transcriptional (iii) regulation of *Foxo1* during B cell progenitor differentiation. (B) Western blotting for FOXO1 protein expression during the Fr.C to Fr.D transition. (C) Analysis of scRNA-seq time series data for a correlation between *Foxo1* mRNA level and the expression of FOXO1 target genes. FOXO1 target genes were defined by ChIP-seq peaks in promoters as described above (black ORs and *P* values). Alternatively, FOXO1 target genes were defined based on the deregulation of genes after genetic deletion of *Foxo1* in early lymphoid/B cell progenitor cells ([40] blue ORs and *P* values). B3 cells with low, medium, and high *Foxo1* mRNA level were analyzed separately (left), cells with low and medium *Foxo1* mRNA level were pooled (middle), or cells with medium and high *Foxo1* mRNA level were pooled (right). Genes were defined as significantly associated with *Foxo1* if *P* < 0.01 (Fisher test). We conducted an enrichment analysis comparing targets and nontargets. *P* values and ORs are shown. (D) Examples for the relationship between *Foxo1* mRNA and mRNA expression of selected FOXO1 targets in scRNA-seq time series data. The source of the numerical data underlying this figure is listed in S1 Data. (E) The bars indicate the amount of variance (r^2^ values calculated based on the difference between linear models) of FOXO1 target gene expression [40] after Ikaros induction by 4-OHT treatment is accounted for by time, *Foxo1* mRNA expression, and the combination of time and *Foxo1* mRNA expression in scRNA-seq. The source of the numerical data underlying this figure is listed in S1 Data. ChIP-seq, chromatin immunoprecipitation sequencing; FOXO1, Forkhead Box O1; Fr.C, proliferating B cell progenitor; Fr.D, differentiating B cell progenitor; OR, odds ratio; scRNA-seq, single cell RNA sequencing; 4-OHT, 4-hydroxy-tamoxifen.

FOXO1 is essential for B cell development, and its role in the progression from B cell progenitor proliferation to B cell progenitor differentiation (i.e., the FR.C to Fr.D transition) has been characterized in exquisite detail [8,9,11,39]. According to current models, Fr.C cells proliferate in response to interleukin-7 (IL-7) receptor signaling, which inactivates FOXO1 via the phosphatidylinositol 3 kinase/Akt kinase/mechanistic target of rapamycin complex 1 (PI3K/Akt/mTOR) axis (Fig 4Ai). Changes in signaling through the IL-7 receptor and/or pre–B cell receptor lead to the post-translational activation and stabilization of FOXO1 protein (Fig 4Aii). FOXO1 then induces the expression of target genes that are critical for B cell progenitor differentiation and include B cell receptor signaling components, the recombinase activating genes *Rag1* and *Rag2*, and the immunoglobulin light chain loci *Igk* and *Igl* (Fig 4Aiii) [8,9,11]. We confirmed that FOXO1 protein expression progressive increased during Ikaros-induced B3 cell differentiation (Fig 4B).

Our finding that *Foxo1* mRNA increases over the time course of B3 cell differentiation and also between the Fr.C to Fr.D B cell progenitor stages in vivo raise a hitherto unanswered question about the role of FOXO1, namely, whether the transcriptional up-regulation we have uncovered contributes to the regulation of FOXO1 target genes in B cell progenitor differentiation. This is difficult to address by population RNA-seq data, because FOXO1 target genes were differentially expressed during the Fr.C to Fr.D transition, concomitant with the increase in *Foxo1* mRNA (*P* < 10^−13^, for FOXO1 ChIP-seq targets versus nontargets, chi-squared test). To address this conundrum, we interrogated scRNA-seq data. We asked whether *Foxo1* mRNA was positively correlated with the expression of FOXO1 target gene transcripts at the single-cell level and to what extent *Foxo1* mRNA was a predictor of FOXO1 target gene expression independently of time. FOXO1 target genes were defined either by ChIP-seq peaks in promoters as described above or, alternatively, as FOXO1-dependent genes that were deregulated after genetic deletion of Foxo1 in early lymphoid/B cell progenitor cells [40]. We found significant correlations between *Foxo1* mRNA and FOXO1 target gene transcripts when comparing cells with low versus intermediate versus high expression of *Foxo1* transcripts (Fig 4C; see Fig 4D for examples).

We next used linear models to compute the extent to which the level of each differentially expressed target gene was explained by time after 4-OHT addition, by *Foxo1* mRNA level, or a combination of time and *Foxo1* mRNA level. Adding *Foxo1* mRNA levels to the models significantly increased the fraction of the variance in FOXO1 target gene expression that could be explained by time after 4-OHT addition alone (Fig 4E). The scRNA-seq analysis indicates that the level of *Foxo1* mRNA expression correlates with the expression of FOXO1 target genes in the same cells.

### Repression of *Myc* is required for the regulation of a subset of Ikaros target genes in B cell progenitors

**Fig 5:**
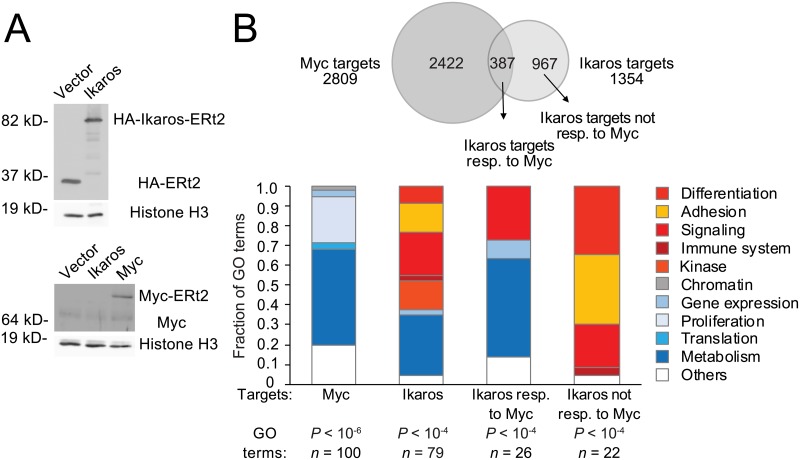
Inducible expression of Myc defines Myc-responsive and Myc-unresponesive Ikaros target genes. (A) Expression of Ikaros-ERt2 and Myc-ERt2 in B3 cells as detected by western blotting. (B) Chromatin RNA-seq showed that the expression of 2,809 genes was regulated by Myc (adj. *P* < 0.05) and the expression of 1,978 genes was regulated by Ikaros (adj. *P* < 0.05) in B3 cells. Of these, 587 genes were responsive to Myc and also to Ikaros (top). GO analysis of the biological function of Myc- and Ikaros-regulated genes, and of Ikaros-regulated genes that are also regulated by Myc (Myc-responsive) or not regulated by Myc (not Myc responsive, bottom). The top GO terms are shown; numbers and *P* values are indicated. The source of the numerical data underlying this figure is listed in S1 Data. ERt2, mutant estrogen receptor; GO, gene ontology; HA, haemagglutinin; Myc, MYC proto-oncogene; RNA-seq, RNA sequencing.

We next examined the regulatory relationship between Ikaros and Myc during B cell progenitor differentiation. We transduced B3 cells with Myc-ERt2 or control vector (Fig 5A). In an attempt to capture immediate Myc target genes in B3 cells, we isolated chromatin-associated RNA [41] for high-throughput sequencing 30, 60, and 180 min after induction with 4-OHT. Analysis over the time series (as described for Fig 1) identified 2,809 differentially expressed genes after Myc induction (adj. *P* < 0.05; 1,485 up, 1,241 down). As a reference, we transduced B3 cells with Ikaros-ERt2 for chromatin-associated RNA-seq as described for Myc-ERt2. Analysis over the time series identified 1,354 differentially expressed genes after Ikaros induction (adj. *P* < 0.05; 662 up, 692 down) (S4 Table and Fig 5B). Gene ontology (GO) analysis of Ikaros-regulated genes showed enrichment of a spectrum of functional terms dominated by adhesion, differentiation, signaling, kinase activity, phosphorylation, and metabolism (Fig 5B). GO term analysis of Myc-regulated genes showed enrichment mainly of metabolism-related terms within the time frame of these experiments (Fig 5B); 387 of the 1,354 Ikaros-regulated genes identified by sequencing of chromatin-associated RNA were also differentially expressed in response to Myc induction, and thus were responsive to regulatory inputs from Myc as well as Ikaros. GO term analysis of these genes showed predominant enrichment of metabolic genes (Fig 5B), similar to Myc-regulated genes. The remaining 967 Ikaros-regulated genes were not differentially expressed in response to Myc induction. GO term analysis of these genes showed enrichment mainly of adhesion, differentiation, the immune system, signaling, kinase activity and phosphorylation (Fig 5B). Therefore, responsiveness to Myc separated Ikaros target genes broadly into those related to metabolism (Myc-responsive) and differentiation (Myc-unresponsive).

**Fig 6:**
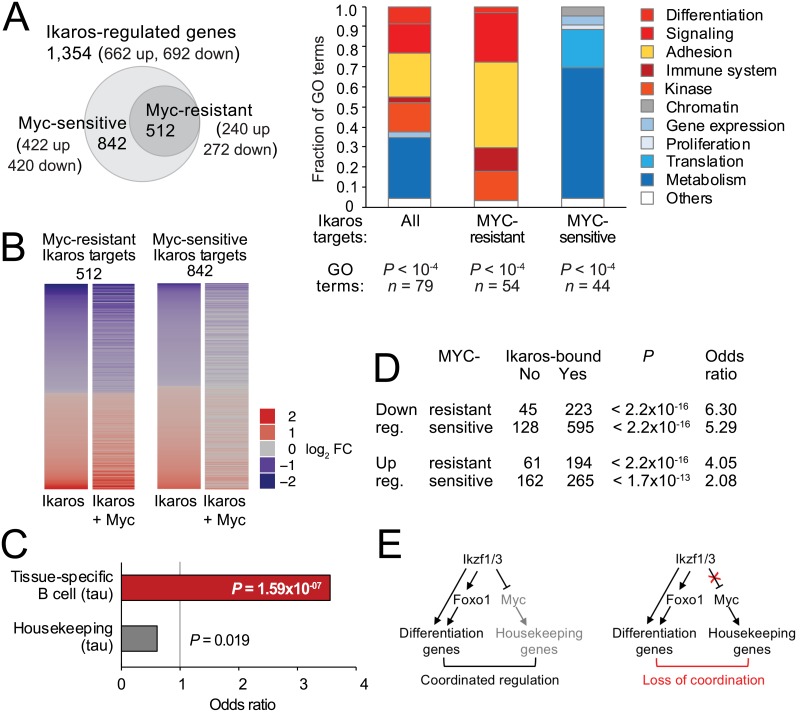
The regulatory relationship between Ikaros and Myc. (A) Of 1,978 genes that were regulated by Ikaros by chromatin RNA-seq (see Fig 5), 582 were also significantly (adj. *P* < 0.05) regulated by Ikaros in the presence of inducible Myc (Myc-resistant Ikaros target genes). The remaining 1,396 Ikaros-regulated genes were Myc sensitive, i.e., not significantly regulated by Ikaros in the presence of Myc (adj. *P* > 0.05). GO analysis of the biological function of differentially expressed genes in response to Ikaros alone or Ikaros + Myc. The top GO terms are shown; numbers and *P* values are indicated. The source of the numerical data underlying this figure is listed in S1 Data. (B) Heat map of log_2_ FCs of Myc-resistant and Myc-sensitive Ikaros target genes in B3 cells. The source of the numerical data underlying this figure is listed in S1 Data. (C) Enrichment of B cell–specific gene sets and depletion of housekeeping genes among Myc-resistant Ikaros targets. Tissue-specific and housekeeping genes were identified based on tau-values compiled from mouse ENCODE RNA-seq data across 22 tissues [43]. (D) Analysis of ChIP-seq data for HA-Ikaros binding at Myc-sensitive and Myc-resistant Ikaros target gene promoters. *P* values were determined by Fisher exact test. Odds ratios > 1 indicate enrichment for Ikaros binding. (E) Coordinated regulation of lineage-specific differentiation genes and housekeeping genes through feedforward regulation of *Myc* (left). Continued *Myc* expression separates the regulation of differentiation and housekeeping genes (right). ChIP-seq, chromatin immunoprecipitation sequencing; ENCODE, encyclopedia of DNA elements; FC, fold change; GO, gene ontology; HA, haemagglutinin; Myc, MYC proto-oncogene; RNA-seq, RNA sequencing.

Ikaros and Aiolos directly bind to the *Myc* promoter and repress *Myc* at the transcriptional level [7,18,42]. Whether repression of *Myc* is essential for coordinating the regulatory roles of Ikaros and Myc in B cell progenitors is currently unknown, although it has been shown that Myc can override the down-regulation of *Ccnd3* (cyclin D3) and the up-regulation of the cell-cycle inhibitor *Cdkn1b* (p27) by Ikaros and Aiolos [42]. These examples indicate that the regulation of at least some Ikaros target genes requires the repression of *Myc*. This raises the question of whether the regulation of target genes by Ikaros generally requires the down-regulation of *Myc* or whether there are classes of Ikaros target genes that do and do not require *Myc* repression. As an experimental system that removes *Myc* from the direct control of Ikaros, we simultaneously transduced B3 cells with both Ikaros-ERt2 and Myc-ERt2 and sequenced chromatin-associated RNA as described above. Of the 1,354 Ikaros target genes identified by induction of Ikaros-ERt2 alone, 512 differentially expressed also in the presence of Myc (240 were up-regulated, and 272 were down-regulated). We refer to these Ikaros target genes as “Myc-resistant” (Fig 6A). The remaining 842 Ikaros target genes were only differentially expressed (adj. *P* < 0.05) in response to Ikaros alone (422 up, 420 down) but were not differentially expressed when Myc was expressed alongside Ikaros (Fig 6A and 6B). We refer to these Ikaros target genes as “Myc-sensitive” (Fig 6A). This analysis defined distinct sets of Ikaros target genes that do or do not require the down-regulation of *Myc*: coexpression of Myc neutralized the impact of Ikaros on Myc-sensitive target genes but not on Myc-resistant target genes (Fig 6B).

GO analysis demonstrated that Myc-resistant Ikaros target genes were enriched for functional terms related to signaling, adhesion, differentiation and development, and the immune system (Fig 6A). By contrast, Myc expression did interfere with Ikaros regulation of target genes related to metabolism, proliferation, and mRNA translation (Fig 6A). To ask whether these differences separated B cell–specific from housekeeping genes, we classified Ikaros-regulated genes during B cell progenitor differentiation as B cell specific or ubiquitously expressed. As a measure for how broadly genes are expressed, we used tau-values compiled from mouse Encyclopedia of DNA Elements (ENCODE) RNA-seq data across 22 tissues [43]. We assembled a panel of broadly expressed genes with tau-values < 0.25 that were expressed > 0.5 fragments per kilobase of exon model per million reads mapped (FPKM) in B3 cells as well as in spleen (an organ rich in B cells), and a panel of tissue-specific genes with tau-values > 0.70 (also expressed > 0.5 FPKM in B3 cells and spleen).

We found that the distribution of tissue-specific and housekeeping genes was skewed between Myc-sensitive and Myc-resistant Ikaros target genes. Housekeeping genes were enriched among Myc-sensitive Ikaros targets (*P* = 0.019, odds ratio = 1.64), and B3 tissue-specific genes were depleted among Myc-sensitive genes (*P* = 2.122 × 10^−5^, odds ratio = 0.42). Conversely, Myc-resistant Ikaros target genes were enriched for B3-specific genes over housekeeping genes (*P* value = 1.59× 10^−7^, odds ratio = 3.57). B3 tissue-specific genes were enriched, and housekeeping genes were depleted among Myc-resistant Ikaros targets (Fig 6C). Validation experiments showed that Myc was able to override Ikaros in the regulation of most glycolysis and glutaminolysis genes (S4A Fig) and substantially reduced the impact of Ikaros on ECAR and OCR in metabolic flux assays (S4B Fig). Consistent with data in primary pre–B cells [42], Myc also prevented Ikaros-imposed cell-cycle exit of B3 cells (S4C Fig). The regulatory relationship between Ikaros and Myc is illustrated by the target genes *Igll1* (dominated by Ikaros), *Ccnd2* (dominated by Myc), and *Slc2a1* (coregulated by Ikaros and Myc; S4D Fig).

Analysis of Ikaros ChIP-seq data showed that promoters that were up- or down-regulated by Ikaros irrespective of Myc showed strong enrichment for Ikaros binding (*P* < 2.2 × 10^−16^, odds ratio = 6.30 and *P* < 2.2 × 10^−16^, odds ratio = 4.05, respectively; Fig 6D). Myc-sensitive Ikaros-regulated genes were also significantly enriched for Ikaros binding (*P* < 2.2 × 10^−16^, odds ratio = 5.92 for down-regulated and *P* < 1.7 × 10^−13^, odds ratio = 2.08 for up-regulated genes compared to all expressed genes; Fig 6D), suggesting that many promoters may receive regulatory inputs from both Ikaros and Myc.

The picture emerging from this analysis is that Ikaros regulates both ubiquitous and tissue-specific genes. Regulation of most ubiquitous genes by Ikaros is broadly sensitive to Myc dosage, whereas regulation of many tissue-specific genes by Ikaros is resistant to Myc dosage. These data are consistent with the existence of a regulatory circuit whereby Ikaros down-regulates *Myc*, and target gene expression reflects the combined effect of Ikaros expression and *Myc* down-regulation. The data suggest a model of progenitor cell differentiation in which the regulation of lineage-specific differentiation genes is coordinated with that of housekeeping genes. In B cell progenitor cell differentiation, such coordination is achieved by feedforward regulation of *Myc* by Ikaros; Fig 6E, left). Continued Myc activity interferes with the regulation of housekeeping functions and abolishes the coordinated regulation of housekeeping and lineage-specific differentiation genes (Fig 6E, right). This model is consistent with the known role of Myc as a major regulator of metabolism, the cell cycle, and RNA transcription and translation [44] and provides a framework for how Ikaros-mediated *Myc* repression contributes to differential gene expression during B cell progenitor differentiation.

## Discussion

To identify transcriptional pathways that drive B cell progenitor differentiation, we developed the TWM algorithm, which identifies coherence between transcription factors downstream of Ikaros induction and their target gene expression over time. This approach pinpointed the transcriptional up-regulation of *Foxo1* up-regulation and the repression of *Myc* as important components of Ikaros-mediated B cell differentiation.

FOXO1 target genes are integral to Fr.D, including immunoglobulin light gene rearrangement [8,9,11]. Previously, several studies have shown that FOXO1 protein is activated and stabilized at the post-translational level during B cell progenitor differentiation [8,9,11]. Here, we demonstrate that the progressive transcriptional up-regulation of *Foxo1* correlates with the expression of FOXO1 target genes at the single cell level. Perturbation experiments will be required to establish the extent to which transcriptional regulation of *Foxo1* is a functional contributor to FOXO1 target gene expression during B cell progenitor differentiation.

The coordination between proliferation, metabolic state, and differentiation is essential for normal development and homeostasis and has been attributed to antagonism between transcription factors that induce tissue-specific gene expression and cyclin-dependent kinases that promote cell-cycle entry [1–3]. By means of perturbation experiments designed to disrupt feedforward repression of *Myc* by Ikaros, we demonstrate that the regulation of lineage-specific differentiation genes can be dissociated from that of ubiquitously expressed genes, simply by uncoupling pathways by which Ikaros acts as an inducer of B cell progenitor differentiation from the repression of *Myc*.

Destabilization of one state and implementation of another has been studied extensively in cellular reprogramming [45–47], and our analysis introduces a similar idea to progenitor cell differentiation. Repression of *Myc* extinguishes key features of the undifferentiated state, and up-regulation of Ikaros family transcription factors—*Foxo1*, and presumably others—promotes a shift to the differentiated state. Consistent with this model, metabolic Myc target genes [48] were mostly repressed (S5B Fig), whereas FOXO1 target genes related to signaling, adhesion, and the immune system were mainly up-regulated (S5B Fig). Reminiscent of the scenario described here, FOXO and Myc control cell proliferation and metabolism in endothelial cells [49]. In B cell progenitors, both *Myc* and *Foxo1* are direct targets of Ikaros, which links the extinction of the Fr.C-like state and establishment of the Fr.D-like state. Integration between cell type–specific and ubiquitous gene expression programs by interconnected regulators may account for other cell-state transitions.

## Methods

### Ethics statement

Mouse work was performed according to the Animals (Scientific Procedures) Act under the authority of project licence PPL70/7556 issued by the Home Office, United Kingdom. All work with mouse cells was in vitro, and the ARRIVE checklist is not applicable.

Cell culture, retroviral transduction, RNA, and protein methods were as described by Ferreirós and colleagues [7]. Isolation of chromatin-associated RNA was done as described by Ma and colleagues [42].

### RNA-seq

To control for sources of variability, we implemented a scheme that tracks biological batches (3), conditions (Ikaros or control vector), time points (6), library preparations (6 × 6), bar codes, sequencing runs, and flow cell lanes. Each RNA-seq library was split into two (total of 72) to account for variability associated with sequencing. For sequencing, the 72 libraries were distributed across 4 flow cells with 3 libraries per flow cell. Each lane contained different libraries, batches, time points, and conditions. We aimed for 50 million reads per library × 4 sequencing runs, equaling 100 million reads per sample. Strand-specific libraries were sequenced on an Illumina HiSeq 2500, at 75 nucleotides paired-end. Analysis followed published guidelines [50] using Tophat2 “very-sensitive” mode only allowing a unique best mapping to map sequences to the mm10 reference genome. Trimming was applied to remove Illumina primers and low-quality nucleotides. ht-seq intersection-option was used to assign fragments to genes. We used cqn to correct for GC content and gene-length bias and a nonparametric version of ComBat to correct for RNA-seq library-preparation effects.

We identified differential expression by combining limma [19] to identify sharp changes and maSigPro [20] to identify changes over time. In limma, we computed significant differences between Ikaros (4-OHT and Ikaros effects) and control (4-OHT effects)—between consecutive time points and between every time point and 0 h. To compute a final limma-derived *P* value for each gene, we combined all contrasts using eBayes function of limma and computed the F-statistic, the associated *P* value, and the adj. *P* value (using Benjamini-Hochberg multiple testing correction). In maSigPro, we considered 2 conditions (Ikaros and control) and identified genes whose trends separated over time. We considered a gene to be differentially expressed if any of the following conditions applied: (a) limma FDR < 0.001, (b) maSigPro R^2^ > 90%, or (c) limma FDR < 0.01 and maSigPro R^2^ > 60%.

### Gene expression microarray analysis

We used limma to analyze gene expression array data from www.immgen.org.

### Gene set analysis

We used Generally Applicable Gene-set Enrichment [51] to perform gene set analysis over ranked lists of genes [52] and Fisher test to perform gene set analysis over clusters of genes. We used default parameters and defined as significant those gene sets with associated Benjamini-Hochberg adj. *P* < 0.1. We used gene sets included in the GSKB Bioconductor package [53].

### TWM

S3 Fig describes the analysis step by step. A mathematical description is given in “Notation.”

### Notation

1. *Time T* = (*x*_1_, *x*_2_, …, *x*_*A*_)
2. $Differences\;between\;consecutive\;times\;\nabla^{T}=\left\{\nabla_{x_{2}-x_{1}},\nabla_{x_{3}-x_{2}},\ldots ,\;\nabla_{x_{A}-x_{A-1}}\right\}$
3. $Consecutive\;Contrasts\;C\nabla^{T}=\left\{\left(\nabla_{x_{2}-x_{1}},\nabla_{x_{3}-x_{2}}\right),\left(\nabla_{x_{3}-x_{2}},\nabla_{x_{4}-x_{3}}\right),\ldots ,\left(\nabla_{x_{A-1}-x_{A-2}},\nabla_{x_{A}-x_{A-1}}\right)\right\}$
4. *Transcription Factors TF* = {*tf*_1_, *tf*_2_, …, *tf*_*F*_}
5. *Genes G* = {*g*_1_, *g*_2_, …, *g*_*Tg*_}
6. $Binding\;Information\;Graph\;BIG=\{TF,\;G,\;b_{{tf}_{j},g_{j}}=\begin{array}{cc} 1 & if\;{tf}_{i}\;binds\;g_{j}\\ 0 & otherwise \end{array}\}$
7. $Differential\;expression\;DEI=\left\{\nabla^{T},\;G,\;dei_{\nabla_{a},g_{j}}=\begin{array}{cc} 1 & if\;g_{j}\;differentially\;expressed\;at\;\nabla_{a}\\ 0 & otherwise \end{array}\right\}$
8. *log*2 *fold-change*: *logarithm* 2 *transformation of the fold change of a given gene*
9. *OR*: *odd ratio quantifying the enrichment of a transcription factor targets over differentially expressed genes*

### Step 1: Log_2_ fold change

1. Computing log_2_ fold change per contrast:
  $$\forall \;{tf}_{i}\;\in TF,\;\forall \;\nabla_{j}\;\in \nabla^{T}\;estimate\;{logFC}_{{tf}_{i},\nabla_{j}}$$
2. Computing aggregated log_2_ fold change for consecutive contrasts:
  $$\forall tf_{i}\in TF,\forall \text{C}\nabla_{j}=(\nabla_{a},\nabla_{b})\in \text{C}\nabla^{T}estimate\;logFC_{tf_{i},\text{C}\nabla_{j}}=logFC_{tf_{i},\nabla_{a}}+logFC_{tf_{i},\nabla_{b}}$$

### Step 2: Odds ratios

1. Computing odds ratios:
  $$\forall \;tf\;\in TF,\;\forall \;\nabla_{j}\in \nabla^{T}\;estimate\;{OR}_{{tf}_{i},\nabla_{j}}$$
2. Computing aggregated log_2_ fold change for consecutive contrasts
  $$\forall tf_{i}\in TF,\forall \text{C}\nabla_{j}=(\nabla_{a},\nabla_{b})\in \text{C}\nabla^{T}estimate\;OR_{tf_{i},\text{C}\nabla_{j}}=OR_{tf_{i},\nabla_{a}}+OR_{tf_{i},\nabla_{b}}$$

### Step 3: Coherence

1. Computing direction for all genes and transcription factors for all contrasts:
  $$\forall \;g\;\in G,\;\forall \;\nabla_{j}\in \nabla^{T}\;estimate\;{sign}_{g,\nabla_{j}}=sign({logFC}_{g,\nabla_{j}})$$
  $$\forall \;{tf}_{i}\;\in TF,\;\forall \;\nabla_{j}\;\in \nabla^{T}\;estimate\;{sign}_{{tf}_{i},\nabla_{j}}=sign({logFC}_{{tf}_{i},\nabla_{j}})$$
2. Computing coherence per transcription factor for each gene and for each consecutive contrast:
  $$\forall \;tf_{i}\in TF,$$
  $$\forall \;\text{C}\nabla_{j}=\left(\nabla_{a},\nabla_{b}\right)\in \text{C}\nabla^{T},$$
  $$\forall \;g\;\in G\;\;|\;\;b_{tf_{i},g}=1\;\wedge \;dei_{\nabla_{a},g}\;=1\;\wedge \;dei_{\nabla_{b},g}\;=1,$$
  $$compute\;coh_{g,tf_{i},\text{C}\nabla_{j}}=\begin{array}{cc} 1 & if\;sign_{g,\nabla_{a}}*sign_{g,\nabla_{b}}*sign_{tf_{i},\nabla_{a}}*sign_{tf_{i},\nabla_{b}}>0\\ 0 & otherwise \end{array}$$
3. Computing coherence per transcription factor per consecutive contrast:
  $$\forall \;tf_{i}\in TF,\;\forall \;\text{C}\nabla_{j}=\left(\nabla_{a},\nabla_{b}\right)\in \text{C}\nabla^{T},\;\forall \;g\;\in G\;\;|\;\;b_{tf_{i},g}=1\;compute$$
  $$compute\;coh_{tf_{i},\text{C}\nabla_{j}}=\;\frac{\sum_{\forall \;g\;\in G\;\;|\;\;b_{tf_{i},g}=1}coh_{g,tf_{i},\text{C}\nabla_{j}}}{\#\;g\;\in G\;\;|\;\;b_{tf_{i},g}=1\;\wedge \;dei_{\nabla_{a},g}\;=1\;\wedge \;dei_{\nabla_{b},g}\;=1}$$

### Step 4: Combine

1. Combine log_2_ fold change and odds ratio measurements:
  $$\forall \;{tf}_{i}\;\in TF,\;{comb}_{{tf}_{i}}=\sum_{\forall \;{C\nabla }_{j}\;\in \;C\nabla \;}{logFC}_{{tf}_{i},{C\nabla }_{j}}{*OR}_{{tf}_{i},{C\nabla }_{j}}$$
2. Combine all coherence:
  $$\forall \;{tf}_{i}\;\in TF,\;{coh}_{{tf}_{i}}=\prod_{\forall \;{C\nabla }_{j}\;\in \;C\nabla \;}{coh}_{{tf}_{i},{C\nabla }_{j}}$$
3. Combine all measurements:
  $$\forall \;{tf}_{i}\;\in TF,\;{TWM}_{{tf}_{i}}={comb}_{{tf}_{i}}*{coh}_{{tf}_{i}}$$

### B-ALL analysis

CEL files for 1404 B-ALL patients were obtained from their respective publications [23,54–57]. The raw data were normalized together with the Robust Median Average (RMA) algorithm, whereas systematic variations across studies were eliminated using ComBat. Differentially expressed genes between Ikaros-mutated and Ikaros wild-type subjects were identified with limma, using surrogate Variable Analysis [58] to account for possible latent confounders. Pathway enrichment analysis was performed using (a) mean-rank gene set enrichment [59] on gene sets from the Molecular Signature Database [52] and (b) GSEA [52] on Ikaros targets identified by Chip-seq in mouse B3 cells. Enrichment analysis was repeated on the 10,111 genes expressed in B3 cells.

### ChIP-seq data bioinformatics analysis

We analyzed ChIP-seq data for B cell–associated transcription factors (S3 Table). EBF1, PU.1, IRF4 [15], PAX5 [60], CTCF [6], and RUNX1 [61] were from pro–B cells; FOXO1 was from pre–B cells [8]; Myc was from CH12 cells [62]; and Ikaros was from B3 cells [7]. We used Bowtie2 [63] for mapping to the mm10 reference genome. When fastq files were available, we used “end-to-end” and “very-sensitive” parameters, otherwise we used “local” parameter. In all cases, we filtered duplicated reads and any reads with quality scores below 20. We applied CLCbio Peak Finder tool [37] to identify peaks for each sample using default parameters; we used control libraries when available (S1 Table). We considered peaks with adj. *P* < 0.01.

### DNase-seq profiling

DNase-seq was performed on 20 to 25 million cells with 3 biological replicates for all time points and conditions. Briefly, cells were harvested and washed with cold 1× PBS. Lysis conditions were optimized to ensure >90% recovery of intact nuclei. Enrichment of DNaseI hypersensitive fragments (0–500 bp) was performed using a low-melt gel size selection protocol. Library preparation was performed and sequenced as 43-bp paired-end NextSeq 500 Illumina reads. DNaseI libraries were sequenced at a minimum depth of 20 million reads per each biological replicate and a total of 200 million per time and condition. DNase-seq reads were trimmed to 36 bp and paired-end mapped to the mm10 reference genome using Bowtie2 [63] with the following options: -v 2 -k 1 -m 1—best–strata. We used Wellington to identify the footprinting sites per time and condition [64]. We used MATCH algorithm from TRANSFAC to predict binding sites (BSs) of transcription factor motifs in FP. To minimize the number of false positive BS predictions we defined an FOS-related optimal threshold by using as a gold-standard the ChIP-seq IKZF1 peaks. We identified the optimal level of FOS [64] at which DNase-seq–derived Ikzf1 BS predictions obtained by MATCH were optimally (minimizing false positives) predicting CHIP-seq IKZF1 peaks. Proximal genes for ChIP-seq–and DNase-seq–derived peaks were identified with default parameters in RGmatch [38].

### scRNA-seq profiling

We isolated cells using the Fluidigm C1 System. Single-cell C1 runs were completed using the smallest IFC (5–10 μm). Cells were collected at a concentration of 400 cells/μl in a total of 50 μl. To optimize cell capture rates on the C1, buoyancy estimates were optimized prior to each run. Single-cell capture efficiency was between 75% and 90% across 8 runs. Each C1 capture site was visually inspected for single-cell capture and cell viability. After visualization, the IFC was loaded with Clontech SMARTer kit lysis, RT, and PCR amplification reagents. After harvesting, cDNA was normalized across all libraries from 0.1 to 0.3 ng/μl, and libraries were constructed using Illumina’s Nextera XT library prep kit per Fluidigm’s protocol. Constructed libraries were multiplexed and purified using AMPure beads. The final multiplexed single-cell library was analyzed on an Agilent 2100 Bioanalyzer for fragment distribution and quantified using Kapa Biosystem’s universal library quantification kit. The library was normalized to 2 nM and sequenced as 75-bp paired-end dual indexed reads using Illumina’s NextSeq 500 system at a depth of approximately 1.0 to 2.0 million reads per library. Each Ikaros time point was performed once, with the exception of 18- and 24-h time points, in which 2 C1 runs were required in order to achieve approximately 50 single-cells per time point. We mapped 560 scRNA-seq libraries with Tophat2 [65] to the mouse Ensembl gene annotations and mm10 reference genome. We excluded single-cell libraries with a mapping rate less than 50% and less than 450,000 mapped reads; we obtained a total of 324 single cells for all subsequent analysis. Cufflinks [66] version 2.2.1 was used to quantify expression from single-cell libraries using Cuffquant. Gene expression data for each single-cell library were merged and normalized into a single data matrix using Cuffnorm. Monocle [21] was used to compute pseudotime trajectories, in which cells are ordered by their actual progress in the differentiation course rather than by their experimental time point.

### Validation of *Foxo1* expression and FOXO1 targets in single cells

We filtered for cells with expression greater than 0 and grouped cells based on quantiles 0.33 and 0.66 of *Foxo1* expression levels (3 levels: low, middle, and high). Then we did the same for each gene. Using both groupings, we computed a contingency table (Fig 6C, left panel) and a *P* value for each gene and defined genes as significantly regulated and not significantly regulated. We found that significantly regulated genes were enriched in FOXO1 targets (*P* = 0.05). We repeated the analysis, first by combining low and middle into a single level (Fig 5C, middle panel, *P* < 0.05) and second by combining middle and high into a single level (Fig 5C, right panel, *P* < 0.07). Finally, we combined all significantly regulated definitions and considered a gene to be *Foxo1* regulated if the contingency table analysis was significant for any of the 3 analyses. In this case, significantly regulated genes were enriched in FOXO1 targets (*P* < 0.001). To quantify and compare the role of time and *Foxo1* mRNA levels in FOXO1 targets, we first filtered for cells with expression greater than 0 for each gene pair. Then we computed 2 linear models: in the first one, mRNA gene expression was predicted using time; in the second, mRNA gene expression was predicted using a 6-level grouping of *Foxo1* mRNA expression. Finally, for each gene we compared the r^2^ values derived from each model.

Measurement of ECAR and OCR was done using Seahorse XF24 or XF96 extracellular flux analyzers as advised by the manufacturers (Seahorse Bioscience; North Billerica, MA). To assess the impact of Ikaros and Aiolos on ECAR and OCR, primary B cells were transduced with IRES-GFP, *Ikzf1*-IRES-GFP, or *Ikzf3*-IRES-GFP; sorted for GFP expression 72 h later; and rested for 3 to 6 h in cultured in the presence of IL-7.

### Categorization of GO terms in broad functional classes

“Immune system” includes the terms “immune,” “host defense,” “B cell,” “T cell,” “myeloid,” “lymphocyte,” “leukocyte,” and “hematopoiesis.” “Signaling” includes the terms “signal,” “signaling,” “response,” “stimulus,” “communication,” and “activation.” “Adhesion” includes the terms “adhesion” and “integrin.” “Differentiation” includes the terms “differentiation” and “development.” “Metabolism” includes the terms “metabolic,” “metabolism,” “biosynthetic,” “biosynthesis,” and “catabolic.” “Translation” includes the terms “translation,” “ribosome,” and “ribonuclear.” “Proliferation” includes the terms “proliferation,” “chromatid,” “spindle,” “mitosis,” “mitotic,” “cell cycle,” “cell division,” “DNA synthesis,” and “DNA replication.”

## Supporting information

S1 Data Underlying data for figures. (XLSX)

S1 Fig Ikaros target genes identified during B cell progenitor differentiation are deregulated in *IKZF1-mutant* B-ALL. We assembled gene expression profiles of 1,404 B-ALL samples with and without *IKZF1* mutations [23,54–57]. *IKZF1* was mutated in 406 samples (29%). B-ALL samples with and without *IKZF1* mutations differed in 7,222 genes (0.1 FDR; S1A Fig) [67,68] with significant enrichment for 317 of 1,415 gene sets in the molecular signature database [69] *(P* < 5 × 10^−4^), including FOXO, Myc, and CXCR4 pathways, adherens junction, cell cycle, integrin-, B cell receptor-, PI3K-, ERK-, MAPK-, and NF-kB signaling pathways, and the PYK2 pathway, which links leukemia with cell adhesion [25] (S5 Table). Among B-ALLs with BCR-ABL1 translocations, 71% had *IKZF1* mutations; 1,228 genes were differentially expressed in *IKZF1* mutated samples (S1B Fig) with enrichment of FOXO, Myc, CXCR4, chemokine signaling, cell cycle, transcription, adherens junction, focal adhesion kinase, integrin, and B cell receptor signaling as well as the downstream transduction pathways PI3K, ERK, MAPK, NF-kB, and NFAT (*P* < 5 × 10^−4^, S6 Table). Differentially expressed genes in *IKZF1*-mutated B-ALL were enriched for Ikaros target genes identified by ChIP-seq in mouse B3 cells (S1C Fig, *P* = 0.0189 for all samples and *P* = 0.0017 for BCR-ABL1 samples). There was significant overlap between differentially expressed genes in *IKZF1-*mutated B-ALL and early Ikaros targets regulated between 0 and 2 h after 4-OHT (Fig 1F, odds ratio = 2.53, adj. *P* = 0.02 for the 200 most differentially expressed genes). Analysis of the BCR-ABL subset of B-ALL samples identified JAK-STAT (S1D–S1F Fig), G-protein coupled receptor and cytokine signaling (S6 Table). Gene-based prognostic models define subgroups of B-ALL with poor clinical outcome [17,28,70], and a set of 139 asparaginase and vincristine resistance genes [70] was enriched for differential expression during the Fr.C to Fr.D transition (*P* < 0.05). A 256-probe set “Ph+like” signature indicative of poor prognosis [17] was significantly enriched among genes differentially expressed at 2, 6, and 12 h (all *P* < 0.05) after nuclear translocation of Ikaros. Combining 2 distinct Ph+like signatures [17,28] resulted in enrichment at all time points (*P* < 0.05). As a control for the overlap in gene expression between Ikaros-induced B3 cells and IKZF1 mutations in B-ALL, we used recurrent non-*IKZF1* genetic lesions in AML, or B-ALL with 4-OHT-treated B3 cells transduced with ERt2 control vector instead of Ikaros-ERt2. Therefore, analysis of B cell progenitor cell state transitions can reveal gene expression signatures with relevance to human disease. (A, B) Differential expression in 1,404 B-ALL samples (A) and of the BCR-ABL1 subset (B). Log_2_ fold change between wild-type and *IKZF1*-mutated samples log10 adj. *P* values are indicated. Dashed line: log_2_ fold change > 0.5; blue: FDR > 0.1. The sources of the numerical data underlying this figure are listed in S1 Data. (C) GSEA of Ikaros-bound genes identified by ChIP-seq in mouse B3 cells in genes differentially expressed in IKZF1-mutated versus nonmutated human B-ALL. The x-axis is the list of genes ordered by magnitude of differential expression, whereas the y-axis represents the enrichment score for the Ikaros target gene set computed by the GSEA method. The red dashed line indicates the maximum reached by the enrichment score. (D) JAK-STAT signaling pathway in B-ALL. (E,F) JAK-STAT signaling pathway changes between 0 h to 2 h (B) and 0 h to 6 h (C) during the Fr.C to Fr.D transition in vitro. No such overlap was seen when contrasting Ikaros-induced B3 cells with recurrent (non-*IKZF1*) genetic lesions in AML, or B-ALL with 4-OHT-treated B3 cells transduced with ERt2 control vector instead of Ikaros-ERt2. adj., adjusted; AML, acute myeloid leukemia; B-ALL, B cell progenitor acute lymphoblastic leukemia; BCR-ABL1, B-ALL with translocations between the IGH locus and the ABL1 proto-oncogene; ChIP-seq, chromatin immunoprecipitation sequencing; CXCR4, C-X-C chemokine receptor type 4; ERK, extracellular signal–regulated kinase; ERt2, mutant estrogen receptor; FDR, false discovery rate; FOXO, Forkhead Box O; Fr.C, proliferating B cell progenitor; Fr.D, differentiating B cell progenitor; GSEA, Gene Set Enrichment Analysis; JAK-STAT, Janus kinase/signal transducers and activators of transcription; MAPK, mitogen activated kinase; Myc, MYC proto-oncogene; NFAT, nuclear factor of activated T cells; NF-kB, nuclear factor kappa-light-chain-enhancer of activated B cells; PI3K, phosphatidylinositol 3 kinase; PYK2, proline-rich tyrosine kinase 2; 4-OHT, 4-hydroxy-tamoxifen.
(PNG)

S2 Fig Reduced mTORC1 activity and expression of autophagy regulators during B cell progenitor differentiation. (A) Ikaros-induced reduction in mTORC1 activity as indicated by the reduced phosphorylation of ribosomal S6 protein on Ser235/236 and Ser240/244. (B) Transcriptional up-regulation of autophagy regulators during the Fr.C to Fr.D transition in vitro and in vivo. The source of the numerical data underlying this figure is listed in S1 Data. Fr.C, proliferating B cell progenitor; Fr.D, differentiating B cell progenitor; mTORC1, mechanistic target of rapamycin complex 1.
(PNG)

S3 Fig Step-by-step account of TWM. TWM ranks TFs by combining 3 sources of information: (i) TF binding to gene promoters in B cell progenitors from publicly available ChIP-seq data, (ii) differential expression within the Ikaros time series between consecutive time points and over the entire time span (0–24 h), and (iii) coherence between the expression of TFs and their targets over time. The approach has 4 steps: (1) For each TF, log_2_ fold change in expression is averaged for each consecutive pair of contrasts (red). (2) Enrichment of TF binding over differentially expressed genes is averaged for each consecutive pair of contrasts (yellow). (3) For each TF, coherence is determined between expression of the TF and its target genes over time (blue). (4) The log_2_ fold change and odds ratio for each pair of contrasts are multiplied to generate a combination matrix (gray, center), and the sum of these values is multiplied with the global coherence score to determine the final TWM score (gray, right). ChIP-seq, chromatin immunoprecipitation sequencing; TF, transcription factor; TWM, transition weight matrix.
(PNG)

S4 Fig Myc partially overrides the impact of Ikaros on metabolic gene expression and function. (A) Interactions between Ikaros and Myc in metabolic gene regulation. *P* values refer to Ikaros versus control vector (left) and Ikaros versus Ikaros + Myc (right). The numerical data underlying this figure are included in S1 Data. (B) Interactions between Ikaros and Myc in the regulation of metabolic functions, ECAD and OCR. *P* values refer to Ikaros versus control vector (left) and Ikaros versus Ikaros + Myc (right). The numerical data underlying this figure are included in S1 Data. (C) Myc overrides Ikaros-imposed cell-cycle arrest in B3 cells. (D) Schematic representation of the regulatory relationships between Ikaros and Myc at selected target genes. The numerical data underlying this figure are included in S1 Data. ECAD, extracellular acidification rate; Myc, MYC proto-oncogene; OCR, oxygen consumption rate.
(PNG)

S5 Fig An updated network of B cell progenitor differentiation. Based on [8], the model incorporates previous [12,42] and current data. Phase 1 is dominated by IL-7 signaling (panel A; blue indicates posttranslational regulation), phase 2 by FOXO1, pre-B cell receptor signaling, and Ikaros (B). Of 21 validated Myc target genes in core metabolism [30], 19 were differentially expressed during the Fr.C to Fr.D transition. Of these, 18 were down-regulated and 1 was up-regulated. Of 2,186 putative FOXO1 target genes defined by FOXO1 promoter binding, 685 were up- and 308 were down-regulated, including genes related to signaling (81 up- and 24 down-regulated), adhesion (31 up- and 6 down-regulated), and the immune system (23 up- and 10 down-regulated). The source of the numerical data underlying this figure is listed in S1 Data. BCR; FOXO1; Fr.C, proliferating B cell progenitor; Fr.D, differentiating B cell progenitor; IL-7, interleukin-7; Myc, MYC proto-oncogene.
(PNG)

S1 Table Differential expression summary in B3 RNA-seq time series. Limma.P.value (Limma.adj.P.Val) denotes the *P* values of the moderated F-statistic test using limma [19]. MaSigProPval and R2 denote the *P* value and the r-squared of the linear model computed using the MaSigpro tool [20]. CONSENSUS_DE is 1 for those genes that were characterized as differentially expressed (see Methods). logFC and Adjp denotes fold change and adjusted *P* value, respectively, for each contrast. RNA-seq, RNA sequencing.
(XLSX)

S2 Table ChIP-seq data sets for Th17 TWM analysis. ChIP-seq, chromatin immunoprecipitation sequencing; Th17, T helper 17; TWM, transition weight matrix.
(XLSX)

S3 Table ChIP-seq data sets selected for the B3 TWM analysis. Mapping, filtering, and peak columns describe the methodology used in each case, dependent on the quality of reads and availability of background samples. Cell column denotes the cell type analyzed. ChIP-seq; TWM, transition weight matrix.
(XLSX)

S4 Table Genes differentially regulated by Ikaros and Myc. Myc.
(XLSX)

S5 Table B-ALL pathway enrichment, all samples. B-ALL, B cell progenitor acute lymphoblastic leukemia.
(XLSX)

S6 Table B-ALL pathway enrichment, BCR-ABL samples. B-ALL, B cell progenitor acute lymphoblastic leukemia; BCR-ABL, B-ALL with translocations between the IGH locus and the ABL1 proto-oncogene.
(XLSX)

## Abbreviations

4-OHT: 4-hydroxy-tamoxifen
adj.: adjusted
B-ALL: B cell progenitor acute lymphoblastic leukemia
BCR: B cell receptor
BCR-ABL1: B-ALL with translocations between the IGH locus and the ABL1 proto-oncogene
BLNK: B cell linker
BS: binding site
CD: cluster of differentiation
ChIP-seq: chromatin immunoprecipitation sequencing
CTCF: CCCTC-binding factor
DNase-seq: DNase I hypersensitive sites sequencing
ECAR: extracellular acidification rate
ENCODE: Encyclopedia of DNA Elements
ERt2: mutant estrogen receptor
Foxo1: Forkhead Box O1
FPKM: fragments per kilobase of exon model per million reads mapped
Fr.C: proliferating B cell progenitor
Fr.D: differentiating B cell progenitor
GO: gene ontology
IL-7: interleukin-7
IRF4: interferon response factor 4
mTORC1: mechanistic target of rapamycin complex 1
Myc: MYC proto-oncogene
OCR: oxygen consumption rate
PI3K: phosphatidylinositol 3 kinase
RMA: robust median average
RORC: RAR-related orphan receptor C
scRNA-seq: single cell RNA sequencing
TCA: Treg cell, regulatory T cell
TWM: transition weight matrix

## References

[1] Ruijtenberg S, van den Heuvel S. Coordinating cell proliferation and differentiation: Antagonism between cell cycle regulators and cell type-specific gene expression. Cell Cycle. Taylor & Francis; 2016;15: 196–212. 10.1080/15384101.2015.1120925 26825227 PMC4825819

[2] Soufi A, Dalton S. Cycling through developmental decisions: how cell cycle dynamics control pluripotency, differentiation and reprogramming. Development. 2016;143: 4301–4311. 10.1242/dev.142075 27899507 PMC5201050

[3] Agathocleous M, Harris WA. Metabolism in physiological cell proliferation and differentiation. Trends Cell Biol. Elsevier Ltd; 2013;23: 484–492. 10.1016/j.tcb.2013.05.004 23756093

[4] Hardy RR, Carmack CE, Shinton S a, Kemp JD, Hayakawa K. Resolution and Characterization of Pro-B and Pre-Pro-B Cell Stages in Normal Mouse Bone Marrow. J Exp Med. 1991;173: 1213–1225. 1827140 10.1084/jem.173.5.1213 PMC2118850

[5] Painter MW, Davis S, Hardy RR, Mathis D, Benoist C. Transcriptomes of the B and T lineages compared by multiplatform microarray profiling. J Immunol. 2011;186: 3047–57. 10.4049/jimmunol.1002695 21307297 PMC3140206

[6] Lin YC, Jhunjhunwala S, Benner C, Heinz S, Welinder E, Mansson R, et al A global network of transcription factors, involving E2A, EBF1 and Foxo1, that orchestrates B cell fate. Nat Immunol. Nature Publishing Group; 2010;11: 635–43. 10.1038/ni.1891 20543837 PMC2896911

[7] Ferreirós Vidal I, Carroll T, Taylor B, Terry A, Liang Z, Bruno L, et al Genome-wide identification of Ikaros targets elucidates its contribution to mouse B cell lineage specification and pre-B cell differentiation. Blood. 2013;121: 1769–1782. 10.1182/blood-2012-08-450114 23303821

[8] Ochiai K, Maienschein-Cline M, Mandal M, Triggs JR, Bertolino E, Sciammas R, et al A self-reinforcing regulatory network triggered by limiting IL-7 activates pre-BCR signaling and differentiation. Nat Immunol. 2012;13: 300–307. 10.1038/ni.2210 22267219 PMC4028049

[9] Clark MR, Mandal M, Ochiai K, Singh H. Orchestrating B cell lymphopoiesis through interplay of IL-7 receptor and pre-B cell receptor signalling. Nat Rev Immunol. Nature Publishing Group; 2014;14: 69–80. 10.1038/nri3570 24378843 PMC4276135

[10] Desiderio S, Lin W, Liz Z. The cell cycle and V(D)J recombination. Curr Top Microbiol Immunol. 1996;217: 45–59. 8787617 10.1007/978-3-642-50140-1_4

[11] Herzog S, Reth M, Jumaa H. Regulation of B-cell proliferation and differentiation by pre-B-cell receptor signalling. Nat Rev Immunol. 2009;9: 195–205. 10.1038/nri2491 19240758

[12] Thompson EC, Cobb BS, Sabbattini P, Meixlsperger S, Parelho V, Liberg D, et al Ikaros DNA-Binding Proteins as Integral Components of B Cell Developmental-Stage-Specific Regulatory Circuits. Immunity. 2007;26: 335–344. 10.1016/j.immuni.2007.02.010 17363301

[13] Heizmann B, Kastner P, Chan S. Ikaros is absolutely required for pre-B cell differentiation by attenuating IL-7 signals. J Exp Med. 2013;210: 2823–32. 10.1084/jem.20131735 24297995 PMC3865471

[14] Joshi I, Yoshida T, Jena N, Qi X, Zhang J, Van Etten R a, et al Loss of Ikaros DNA-binding function confers integrin-dependent survival on pre-B cells and progression to acute lymphoblastic leukemia. Nat Immunol. 2014;15: 294–304. 10.1038/ni.2821 24509510 PMC4494688

[15] Schwickert TA, Tagoh H, Gültekin S, Dakic A, Axelsson E, Minnich M, et al Stage-specific control of early B cell development by the transcription factor Ikaros. Nat Immunol. 2014;15: 283–93. 10.1038/ni.2828 24509509 PMC5790181

[16] Mullighan CG, Miller CB, Radtke I, Phillips L a, Dalton J, Ma J, et al BCR-ABL1 lymphoblastic leukaemia is characterized by the deletion of Ikaros. Nature. 2008;453: 110–4. 10.1038/nature06866 18408710

[17] Mullighan CG, Xiaoping Su PD, Jinghui Zhang PD, Ina Radtke PD, Letha A.A. Phillips BS, Christopher B. Miller BS, et al Deletion of IKZF1 and Prognosis in Acute Lymphoblastic Leukemia. N Engl J Med. 2009;360: 470–480. 10.1056/NEJMoa0808253 19129520 PMC2674612

[18] Liang Z, Brown KE, Carroll T, Taylor B, Vidal IF, Hendrich B, et al A high-resolution map of transcriptional repression. Elife. 2017;6: 1–24. 10.7554/eLife.22767 28318487 PMC5373822

[19] Law CW, Chen Y, Shi W, Smyth GK. Voom: precision weights unlock linear model analysis tools for RNA-seq read counts. Genome Biol. 2014;15: R29 10.1186/gb-2014-15-2-r29 24485249 PMC4053721

[20] Conesa A, Nueda MJJ, Ferrer A, Talón M. maSigPro: a method to identify significantly differential expression profiles in time-course microarray experiments. Bioinformatics. 2006;22: 1096–102. 10.1093/bioinformatics/btl056 16481333

[21] Trapnell C, Cacchiarelli D, Grimsby J, Pokharel P, Li S, Morse M, et al The dynamics and regulators of cell fate decisions are revealed by pseudotemporal ordering of single cells. Nat Biotechnol. Nature Publishing Group; 2014;32: 381–6. 10.1038/nbt.2859 24658644 PMC4122333

[22] Fraley C, Raftery AE. Enhanced Model-Based Clustering, Density Estimation, and Discriminant Analysis Software: MCLUST. J Classif. 2003;20: 263–286. 10.1007/s00357-003-0015-3

[23] Vitanza NA, Zaky W, Blum R, Meyer JA, Wang J, Bhatla T, et al Prognosis in children with rhabdomyosarcoma: A report of the intergroup rhabdomyosarcoma studies I and II. Pediatr Blood Cancer. 2014;61: 1779–1785. 24976218 10.1002/pbc.25119 PMC4217284

[24] Iacobucci I, Iraci N, Messina M, Lonetti A, Chiaretti S, Valli E, et al IKAROS deletions dictate a unique gene expression signature in patients with adult B-cell acute lymphoblastic Leukemia. PLoS ONE. 2012;7 10.1371/journal.pone.0040934 22848414 PMC3405023

[25] Churchman ML, Mullighan CG. Ikaros: exploiting and targeting the hematopoietic stem cell niche in B-progenitor acute lymphoblastic leukemia. Exp Hematol. ISEH—International Society for Experimental Hematology; 2016;46: 1–8. 10.1016/j.exphem.2016.11.002 27865806 PMC5241204

[26] Churchman ML, Low J, Qu C, Paietta EM, Kasper LH, Chang Y, et al Efficacy of Retinoids in IKZF1-Mutated BCR-ABL1 Acute Lymphoblastic Leukemia. Cancer Cell. Elsevier Inc.; 2015;28: 343–356. 10.1016/j.ccell.2015.07.016 26321221 PMC4573904

[27] Churchman ML, Evans K, Richmond J, Robbins A, Jones L, Shapiro IM, et al Synergism of FAK and tyrosine kinase inhibition in Ph+ B-ALL. JCI Insight. 2016;1: 1–13. 10.1172/jci.insight.86082 27123491 PMC4844070

[28] Den Boer ML, van Slegtenhorst M, De Menezes RX, Cheok MH, Buijs-Gladdines JG, Peters ST, et al A subtype of childhood acute lymphoblastic leukaemia with poor treatment outcome: a genome-wide classification study. Lancet Oncol. 2009;10: 125–134. 10.1016/S1470-2045(08)70339-5 19138562 PMC2707020

[29] Meixlsperger S, Köhler F, Wossning T, Reppel M, Müschen M, Jumaa H. Conventional Light Chains Inhibit the Autonomous Signaling Capacity of the B Cell Receptor. Immunity. 2007;26: 323–333. 10.1016/j.immuni.2007.01.012 17331747

[30] Wang R, Dillon CP, Shi LZ, Milasta S, Carter R, Finkelstein D, et al The Transcription Factor Myc Controls Metabolic Reprogramming upon T Lymphocyte Activation. Immunity. Elsevier Inc.; 2011;35: 871–882. 10.1016/j.immuni.2011.09.021 22195744 PMC3248798

[31] O’Neill LAJ, Kishton RJ, Rathmell J. A guide to immunometabolism for immunologists. Nat Rev Immunol. 2016;16: 553–65. 10.1038/nri.2016.70 27396447 PMC5001910

[32] Ciofani M, Madar A, Galan C, Sellars M, Mace K, Pauli F, et al A Validated Regulatory Network for Th17 Cell Specification. Cell. Elsevier Inc.; 2012;151: 1–15. 10.1016/j.cell.2012.09.016 23021777 PMC3503487

[33] Yosef N, Shalek AK, Gaublomme JT, Jin H, Lee Y, Awasthi A, et al Dynamic regulatory network controlling TH17 cell differentiation. Nature. Nature Publishing Group; 2013;496: 461–8. 10.1038/nature11981 23467089 PMC3637864

[34] Tanaka S, Suto A, Iwamoto T, Kashiwakuma D, Kagami S, Suzuki K, et al Sox5 and c-Maf cooperatively induce Th17 cell differentiation via RORγt induction as downstream targets of Stat3. J Exp Med. 2014;211: 1857–1874. 10.1084/jem.20130791 25073789 PMC4144730

[35] Dang E V., Barbi J, Yang HY, Jinasena D, Yu H, Zheng Y, et al Control of Th17/Treg balance by hypoxia-inducible factor 1. Cell. Elsevier Inc.; 2011;146: 772–784. 10.1016/j.cell.2011.07.033 21871655 PMC3387678

[36] Shi LZ, Wang R, Huang G, Vogel P, Neale G, Green DR, et al HIF1α–dependent glycolytic pathway orchestrates a metabolic checkpoint for the differentiation of Th17 and Treg cells. J Exp Med. 2011;208: 1367–1376. 10.1084/jem.20110278 21708926 PMC3135370

[37] Strino F, Lappe M. Identifying peaks in *-seq data using shape information. BMC Bioinformatics. 2016;17: S206 10.1186/s12859-016-1042-5 27295177 PMC4905608

[38] Furió-Tarí P, Conesa A, Tarazona S. RGmatch: matching genomic regions to proximal genes in omics data integration. BMC Bioinformatics. BMC Bioinformatics; 2016;17: 1–10. 28185573 10.1186/s12859-016-1293-1 PMC5133492

[39] Fistonich C, Zehentmeier S, Bednarski JJ, Miao R, Schjerven H, Sleckman BP, et al Cell circuits between B cell progenitors and IL-7+ mesenchymal progenitor cells control B cell development. J Exp Med. 2018;215: jem.20180778. 10.1084/jem.20180778 30158115 PMC6170173

[40] Mansson R, Welinder E, Ahsberg J, Lin YC, Benner C, Glass CK, et al Positive intergenic feedback circuitry, involving EBF1 and FOXO1, orchestrates B-cell fate. Proc Natl Acad Sci. National Academy of Sciences; 2012;109: 21028–21033. 10.1073/pnas.1211427109 23213261 PMC3529039

[41] Bhatt DM, Pandya-Jones A, Tong AJ, Barozzi I, Lissner MM, Natoli G, et al Transcript dynamics of proinflammatory genes revealed by sequence analysis of subcellular RNA fractions. Cell. 2012;150: 279–290. 10.1016/j.cell.2012.05.043 22817891 PMC3405548

[42] Ma S, Pathak S, Mandal M, Trinh L, Clark MR, Lu R. Ikaros and Aiolos Inhibit Pre-B-Cell Proliferation by Directly Suppressing c-Myc Expression. Mol Cell Biol. 2010;30: 4149–4158. 10.1128/MCB.00224-10 20566697 PMC2937562

[43] Kryuchkova-Mostacci N, Robinson-Rechavi M. Tissue-Specificity of Gene Expression Diverges Slowly between Orthologs, and Rapidly between Paralogs. PLoS Comput Biol 2016;12: 1–13. 10.1371/journal.pcbi.1005274 28030541 PMC5193323

[44] Kress TR, Sabò A, Amati B. MYC: connecting selective transcriptional control to global RNA production. Nat Rev Cancer. Nature Publishing Group; 2015;15: 593–607. 10.1038/nrc3984 26383138

[45] Terranova R, Pereira CF, Du Roure C, Merkenschlager M, Fisher AG. Acquisition and extinction of gene expression programs are separable events in heterokaryon reprogramming. J Cell Sci. 2006;119: 2065–72. 10.1242/jcs.02945 16638804

[46] Treutlein B, Lee QY, Camp JG, Mall M, Koh W, Shariati SAM, et al Dissecting direct reprogramming from fibroblast to neuron using single-cell RNA-seq. Nature. Nature Publishing Group; 2016;534: 391–5. 10.1038/nature18323 27281220 PMC4928860

[47] Chronis C, Fiziev P, Papp B, Butz S, Bonora G, Sabri S, et al Cooperative Binding of Transcription Factors Orchestrates Reprogramming. Cell. Elsevier; 2017;168: 1–18. 10.1016/j.cell.2016.12.016 28111071 PMC5302508

[48] Wang Y-H, Israelsen WJ, Lee D, Yu VWC, Jeanson NT, Clish CB, et al Cell-State-Specific Metabolic Dependency in Hematopoiesis and Leukemogenesis. Cell. Elsevier Inc.; 2014;158: 1309–1323. 10.1016/j.cell.2014.07.048 25215489 PMC4197056

[49] Wilhelm K, Happel K, Eelen G, Schoors S, Oellerich MF, Lim R, et al FOXO1 couples metabolic activity and growth state in the vascular endothelium. Nature. Nature Publishing Group; 2016;529: 1–18. 10.1038/nature16498 26735015 PMC5380221

[50] Conesa A, Madrigal P, Tarazona S, Gomez-Cabrero D, Cervera A, McPherson A, et al A survey of best practices for RNA-seq data analysis. Genome Biol. 2016;17: 13 10.1186/s13059-016-0881-8 26813401 PMC4728800

[51] Luo W, Friedman MS, Shedden K, Hankenson KD, Woolf PJ. GAGE: generally applicable gene set enrichment for pathway analysis. BMC Bioinformatics. 2009;10: 161 10.1186/1471-2105-10-161 19473525 PMC2696452

[52] Subramanian A, Tamayo P, Mootha VK, Mukherjee S, Ebert BL, Gillette M a, et al Gene set enrichment analysis: A knowledge-based approach for interpreting genome-wide. PNAS. 2005;102: 15545–15550. 10.1073/pnas.0506580102 16199517 PMC1239896

[53] Bares V, Ge X. gskb: Gene Set data for pathway analysis in mouse. In: R package version 1.6.1. 2015.

[54] Bungaro S, Dell’Orto MC, Zangrando A, Basso D, Gorletta T, Nigro L Lo, et al Integration of Genomic and Gene Expression Data of Childhood ALLWithout Known Aberrations Identifies Subgroups with Specific Genetic Hallmarks. Genes Chromosomes Cancer. 2009;48: 22–38. 10.1002/gcc.20616 18803328

[55] Harvey RC, Mullighan CG, Wang X, Dobbin KK, Davidson GS, Bedrick EJ, et al Identification of novel cluster groups in pediatric high-risk B-precursor acute lymphoblastic leukemia with gene expression profiling: correlation with genome-wide DNA copy number alterations, clinical characteristics, and outcome Identification of nov. Blood. 2010;116: 4874–4884. 10.1182/blood-2009-08-239681 20699438 PMC3321747

[56] Van Der Veer A, Waanders E, Pieters R, Willemse ME, Van Reijmersdal S V., Russell LJ, et al Independent prognostic value of BCR-ABL1-like signature and IKZF1 deletion, but not high CRLF2 expression, in children with B-cell precursor ALL. Blood. 2013;122: 2622–2629. 10.1182/blood-2012-10-462358 23974192 PMC3795461

[57] Roberts KG, Li Y, Payne-Turner D, Harvey RC, Yang Y-L, Pei D, et al Targetable kinase-activating lesions in Ph-like acute lymphoblastic leukemia. N Engl J Med. 2014;371: 1005–15. 10.1056/NEJMoa1403088 25207766 PMC4191900

[58] Leek JT, Johnson WE, Parker HS, Jaffe AE, Storey JD. The sva package for removing batch effects and other unwanted variation in high-throughput experiments. Bioinformatics. 2012;28: 882–3. 10.1093/bioinformatics/bts034 22257669 PMC3307112

[59] Michaud J, Simpson KM, Escher R, Buchet-Poyau K, Beissbarth T, Carmichael C, et al Integrative analysis of RUNX1 downstream pathways and target genes. BMC Genomics. 2008;9: 363 10.1186/1471-2164-9-363 18671852 PMC2529319

[60] Revilla-I-Domingo R, Bilic I, Vilagos B, Tagoh H, Ebert A, Tamir IM, et al The B-cell identity factor Pax5 regulates distinct transcriptional programmes in early and late B lymphopoiesis. EMBO J. Nature Publishing Group; 2012;31: 3130–46. 10.1038/emboj.2012.155 22669466 PMC3400013

[61] Niebuhr B, Kriebitzsch N, Fischer M, Behrens K, Gunther T, Alawi M, et al Runx1 is essential at two stages of early murine B-cell development Birte. Blood. 2013;122: 413–423. 10.1182/blood-2013-01-480244 23704093

[62] Ecker JR, Bickmore WA, Barroso I, Pritchard JK, Gilad Y, Segal E. ENCODE explained. Nature. 2012;489: 52–55. 10.1038/489052a 22955614

[63] Langmead B, Salzberg SL. Fast gapped-read alignment with Bowtie 2. Nat Methods. 2012;9: 357–360. 10.1038/nmeth.1923 22388286 PMC3322381

[64] Piper J, Elze MC, Cauchy P, Cockerill PN, Bonifer C, Ott S. Wellington: A novel method for the accurate identification of digital genomic footprints from DNase-seq data. Nucleic Acids Res. 2013;41 10.1093/nar/gkt850 24071585 PMC3834841

[65] Kim D, Pertea G, Trapnell C, Pimentel H, Kelley R, Salzberg SL. TopHat2: accurate alignment of transcriptomes in the presence of insertions, deletions and gene fusions. Genome Biol. 2013;14: R36 10.1186/gb-2013-14-4-r36 23618408 PMC4053844

[66] Trapnell C, Roberts A, Goff L, Pertea G, Kim D, Kelley DR, et al Differential gene and transcript expression analysis of RNA-seq experiments with TopHat and Cufflinks. Nat Protoc. Nature Publishing Group; 2012;7: 562–78. 10.1038/nprot.2012.016 22383036 PMC3334321

[67] Witkowski MT, Hu Y, Roberts KG, Boer JM, Mckenzie MD, Liu GJ, Le Grice OD, Tremblay CS, Ghisi M, Willson TA, Horstmann MA, Aifantis I, Cimmino L, Frietze S, Den Boer ML, Mullighan CG, Smyth GK, Dickins RA. Conserved Ikaros-regulated genes associated with B-progenitor acute lymphoblastic leukemia outcome. J Exp Med. 2017;214:773–791. 10.1084/jem.20160048 28190000 PMC5339666

[68] Schjerven H, Ayongaba EF, Aghajanirefah A, Mclaughlin J, Cheng D, Geng H, Boyd JR, Eggesbø LM, Lindeman I, Heath JL, Park E, Witte ON, Smale ST, Frietze S, Müschen M. Genetic analysis of Ikaros target genes and tumor suppressor function in BCR-ABL1 + pre–B ALL. J. Exp. Med. 2017; 214: 793–814. 10.1084/jem.20160049 28190001 PMC5339667

[69] Liberzon A, Subramanian A, Pinchback R, Thorvaldsdóttir H, Tamayo P, Mesirov JP. Molecular signatures database (MSigDB) 3.0. Bioinformatics 2011; 27: 1739–1740 10.1093/bioinformatics/btr260 21546393 PMC3106198

[70] Lugthart S, Cheok MH, Den Boer ML, Yang W, Holleman A, Cheng C, Pui CH, Relling M V., Janka-Schaub GE, Pieters R, Evans WE. Identification of genes associated with chemotherapy crossresistance and treatment response in childhood acute lymphoblastic leukemia. Cancer Cell 2005; 7: 375–386 10.1016/j.ccr.2005.03.002 15837626

