# Supplementary figures and images for: Feedforward regulation of Myc coordinates lineage-specific with housekeeping gene expression during B cell progenitor cell differentiation
Source: PLoS Biol. 2019 Apr 12;17(4):e2006506. doi: 10.1371/journal.pbio.2006506 (PMC6481923; doi:10.1371/journal.pbio.2006506)

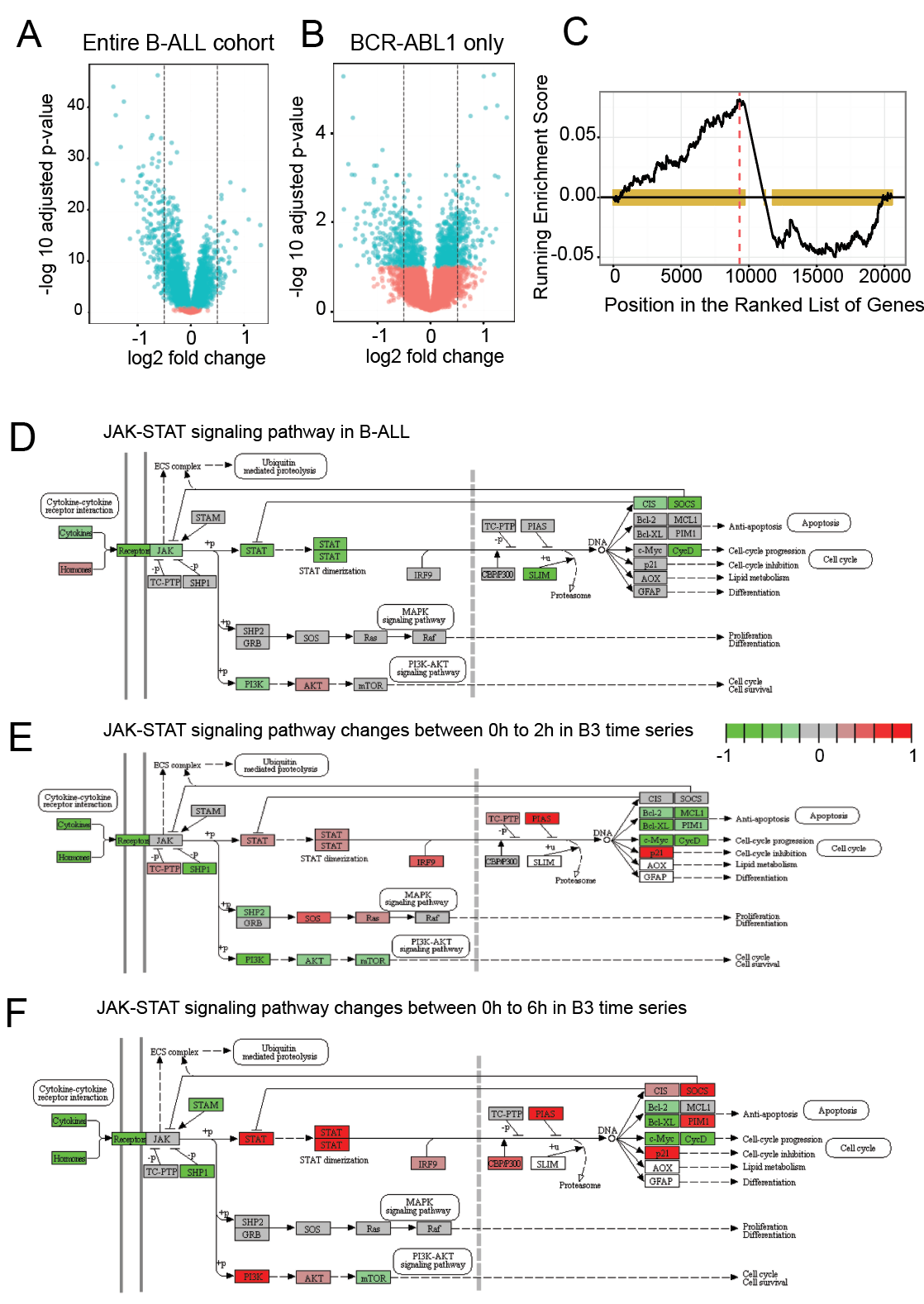

Supplement: S1 Fig — We assembled gene expression profiles of 1,404 B-ALL samples with and without IKZF1 mutations [23,54–57]. IKZF1 was mutated in 406 samples (29%). B-ALL samples with and without IKZF1 mutations differed in 7,222 genes (0.1 FDR; S1A Fig) [67,68] with significant enrichment for 317 of 1,415 gene sets in the molecular signature database [69] (P < 5 × 10^−4^), including FOXO, Myc, and CXCR4 pathways, adherens junction, cell cycle, integrin-, B cell receptor-, PI3K-, ERK-, MAPK-, and NF-kB signaling pathways, and the PYK2 pathway, which links leukemia with cell adhesion [25] (S5 Table). Among B-ALLs with BCR-ABL1 translocations, 71% had IKZF1 mutations; 1,228 genes were differentially expressed in IKZF1 mutated samples (S1B Fig) with enrichment of FOXO, Myc, CXCR4, chemokine signaling, cell cycle, transcription, adherens junction, focal adhesion kinase, integrin, and B cell receptor signaling as well as the downstream transduction pathways PI3K, ERK, MAPK, NF-kB, and NFAT (P < 5 × 10^−4^, S6 Table). Differentially expressed genes in IKZF1-mutated B-ALL were enriched for Ikaros target genes identified by ChIP-seq in mouse B3 cells (S1C Fig, P = 0.0189 for all samples and P = 0.0017 for BCR-ABL1 samples). There was significant overlap between differentially expressed genes in IKZF1-mutated B-ALL and early Ikaros targets regulated between 0 and 2 h after 4-OHT (Fig 1F, odds ratio = 2.53, adj. P = 0.02 for the 200 most differentially expressed genes). Analysis of the BCR-ABL subset of B-ALL samples identified JAK-STAT (S1D–S1F Fig), G-protein coupled receptor and cytokine signaling (S6 Table). Gene-based prognostic models define subgroups of B-ALL with poor clinical outcome [17,28,70], and a set of 139 asparaginase and vincristine resistance genes [70] was enriched for differential expression during the Fr.C to Fr.D transition (P < 0.05). A 256-probe set “Ph+like” signature indicative of poor prognosis [17] was significantly enriched among genes differentially expressed at 2, 6, [file pbio.2006506.s002.png]

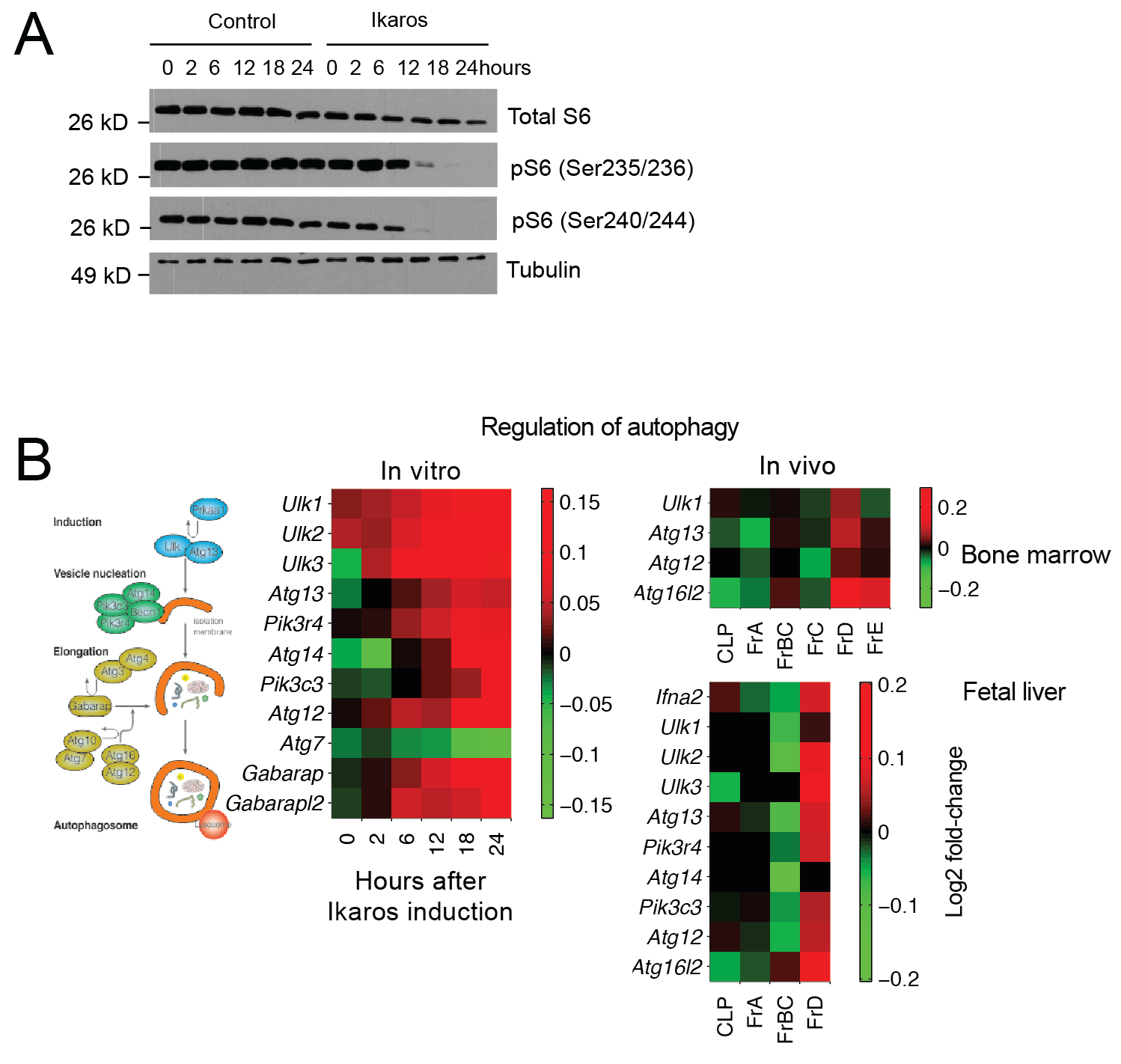

Supplement: S2 Fig — (A) Ikaros-induced reduction in mTORC1 activity as indicated by the reduced phosphorylation of ribosomal S6 protein on Ser235/236 and Ser240/244. (B) Transcriptional up-regulation of autophagy regulators during the Fr.C to Fr.D transition in vitro and in vivo. The source of the numerical data underlying this figure is listed in S1 Data. Fr.C, proliferating B cell progenitor; Fr.D, differentiating B cell progenitor; mTORC1, mechanistic target of rapamycin complex 1. (PNG) [file pbio.2006506.s003.png]

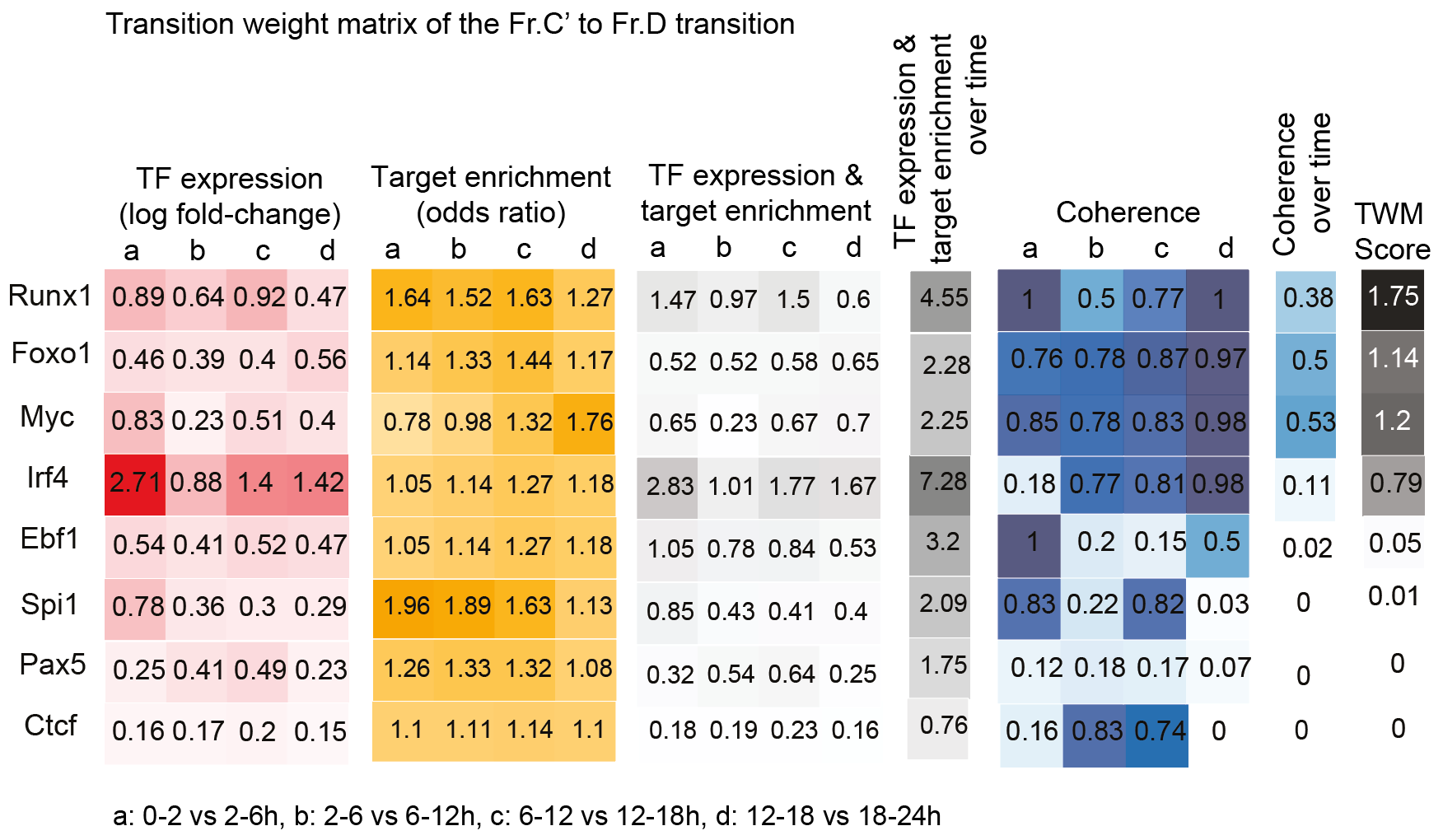

Supplement: S3 Fig — TWM ranks TFs by combining 3 sources of information: (i) TF binding to gene promoters in B cell progenitors from publicly available ChIP-seq data, (ii) differential expression within the Ikaros time series between consecutive time points and over the entire time span (0–24 h), and (iii) coherence between the expression of TFs and their targets over time. The approach has 4 steps: (1) For each TF, log2 fold change in expression is averaged for each consecutive pair of contrasts (red). (2) Enrichment of TF binding over differentially expressed genes is averaged for each consecutive pair of contrasts (yellow). (3) For each TF, coherence is determined between expression of the TF and its target genes over time (blue). (4) The log2 fold change and odds ratio for each pair of contrasts are multiplied to generate a combination matrix (gray, center), and the sum of these values is multiplied with the global coherence score to determine the final TWM score (gray, right). ChIP-seq, chromatin immunoprecipitation sequencing; TF, transcription factor; TWM, transition weight matrix. (PNG) [file pbio.2006506.s004.png]

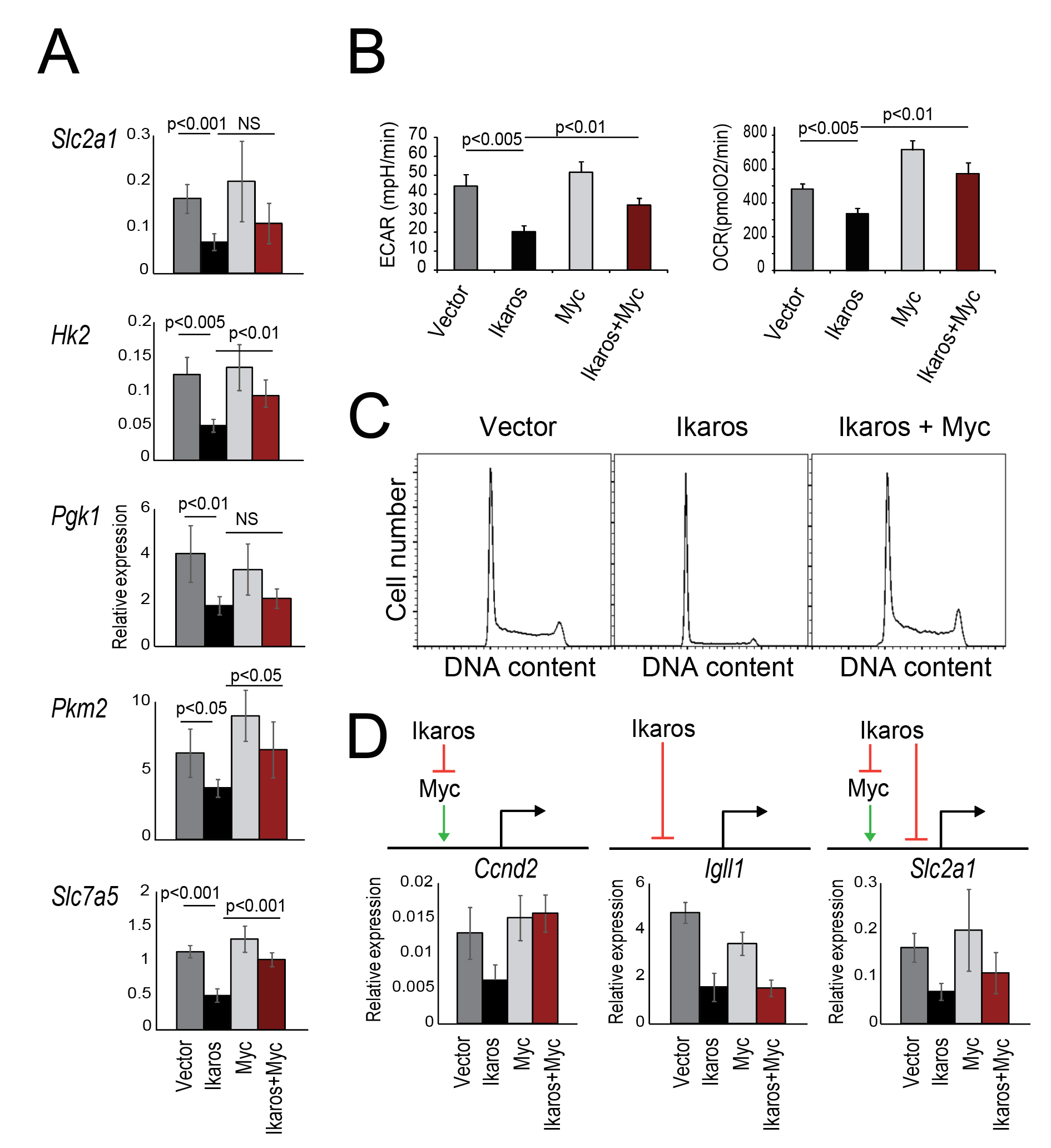

Supplement: S4 Fig — (A) Interactions between Ikaros and Myc in metabolic gene regulation. P values refer to Ikaros versus control vector (left) and Ikaros versus Ikaros + Myc (right). The numerical data underlying this figure are included in S1 Data. (B) Interactions between Ikaros and Myc in the regulation of metabolic functions, ECAD and OCR. P values refer to Ikaros versus control vector (left) and Ikaros versus Ikaros + Myc (right). The numerical data underlying this figure are included in S1 Data. (C) Myc overrides Ikaros-imposed cell-cycle arrest in B3 cells. (D) Schematic representation of the regulatory relationships between Ikaros and Myc at selected target genes. The numerical data underlying this figure are included in S1 Data. ECAD, extracellular acidification rate; Myc, MYC proto-oncogene; OCR, oxygen consumption rate. (PNG) [file pbio.2006506.s005.png]

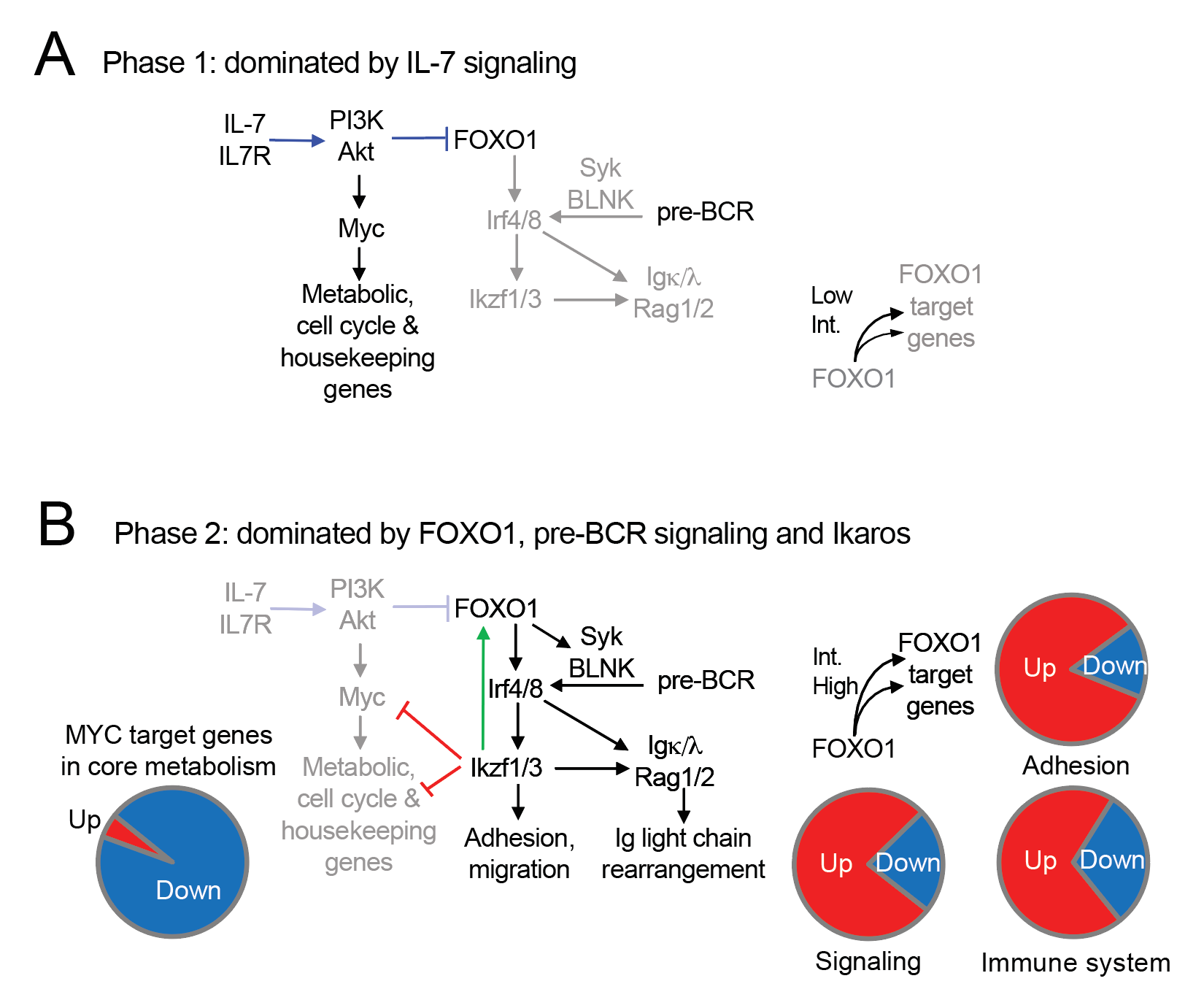

Supplement: S5 Fig — Based on [8], the model incorporates previous [12,42] and current data. Phase 1 is dominated by IL-7 signaling (panel A; blue indicates posttranslational regulation), phase 2 by FOXO1, pre-B cell receptor signaling, and Ikaros (B). Of 21 validated Myc target genes in core metabolism [30], 19 were differentially expressed during the Fr.C to Fr.D transition. Of these, 18 were down-regulated and 1 was up-regulated. Of 2,186 putative FOXO1 target genes defined by FOXO1 promoter binding, 685 were up- and 308 were down-regulated, including genes related to signaling (81 up- and 24 down-regulated), adhesion (31 up- and 6 down-regulated), and the immune system (23 up- and 10 down-regulated). The source of the numerical data underlying this figure is listed in S1 Data. BCR; FOXO1; Fr.C, proliferating B cell progenitor; Fr.D, differentiating B cell progenitor; IL-7, interleukin-7; Myc, MYC proto-oncogene. (PNG) [file pbio.2006506.s006.png]
